# Stress-responsive Gdf15 counteracts renointestinal toxicity via autophagic and microbiota reprogramming

**DOI:** 10.1038/s42003-023-04965-1

**Published:** 2023-06-03

**Authors:** Navin Ray, Seung Jun Park, Hoyung Jung, Juil Kim, Tamas Korcsmaros, Yuseok Moon

**Affiliations:** 1 Laboratory of Mucosal Exposome and Biomodulation, Department of Integrative Biomedical Sciences, Pusan National University, Yangsan, Korea; 2 Division of Digestive Diseases, Faculty of Medicine, Imperial College London, London, UK; 3 Earlham Institute, Norwich Research Park, Norwich, UK; 4 Quadram Institute Bioscience, Norwich Research Park, Norwich, UK; 5 Graduate Program of Genomic Data Sciences, Pusan National University, Yangsan, Korea

**Keywords:** Toxin-induced nephropathy, Transforming growth factor beta

## Abstract

The integrated stress response (ISR) plays a pivotal role in the cellular stress response, primarily through global translational arrest and the upregulation of cellular adaptation-linked molecules. Growth differentiation factor 15 (Gdf15) is a potent stress-responsive biomarker of clinical inflammatory and metabolic distress in various types of diseases. Herein, we assess whether ISR-driven cellular stress contributes to pathophysiological outcomes by modulating Gdf15. Clinical transcriptome analysis demonstrates that PKR is positively associated with Gdf15 expression in patients with renal injury. Gdf15 expression is dependent on protein kinase R (PKR)-linked ISR during acute renointestinal distress in mice and genetic ablation of Gdf15 aggravates chemical-induced lesions in renal tissues and the gut barrier. An in-depth evaluation of the gut microbiota indicates that Gdf15 is associated with the abundance of mucin metabolism-linked bacteria and their enzymes. Moreover, stress-responsive Gdf15 facilitates mucin production and cellular survival via the reorganization of the autophagy regulatory network. Collectively, ISR-activated Gdf15 counteracts pathological processes via the protective reprogramming of the autophagic network and microbial community, thereby providing robust predictive biomarkers and interventions against renointestinal distress.

## Abstract (web summary)

Stress-responsive Growth Differentiation Factor 15 controls acute renointestinal distress by facilitating mucin production and cellular protection via reorganization of cell-protective autophagic signalling and muco-regulatory microbiota in mice.

## Introduction

Renointestinal distress is a pathological outcome of detrimental interorgan communication between the gut and kidney. Accumulating evidence suggests that the gastrointestinal tract is a major source of microbes and immune-related cells, leading to inflammatory insults in patients with acute kidney injury (AKI) or chronic kidney disease (CKD)^1–3^. Likewise, renal manifestations, including renal tubular injury, nephrolithiasis, tubulointerstitial nephritis, glomerulonephritis, and amyloidosis, have been reported in 4–23% of patients with gut barrier distress, such as inflammatory bowel disease^4–6^. Experimentally, it has been shown that advanced CKD can trigger intestinal mucosal barrier damage and subsequent systemic inflammation, thereby aggravating disease severity^3^. In particular, the levels of bacteremia or endotoxemia are highly associated with disease severity in patients with CKD^7,8^. Gut-derived adverse factors, including detrimental microbiota, endotoxins, and uremic toxins, contribute to renal injury^9^. Another representative clinical model of renointestinal distress is a chemotherapy-induced complication^10,11^, although platinum-based chemotherapeutic agents, such as cisplatin (CP), are extensively used to treat solid tumors and hematological malignancies^12^. According to a 6-year cohort investigation, approximately 34% of adult patients with diverse types of cancer administered CP chemotherapy were found to experience AKI, with most patients exhibiting long-term renal outcomes such as a slight but permanent decline in the estimated glomerular filtration rate (eGFR)^13^. In addition to renal injuries, chemotherapy has been associated with mucositis induction^14^. Despite the beneficial effects of anticancer agents, 20–40% of patients with solid tumors reportedly develop mucositis following anticancer therapy^15^. Rapidly growing cells or tissues, such as the mucous membranes of the mouth, stomach, and intestines, are primary targets of detrimental actions mediated by anticancer drugs via genotoxic stressors^16^.

Growth differentiation factor 15 (Gdf15) is a member of the transforming growth factor (TGF)-β superfamily and is induced by diverse pathological stimuli, including tissue injuries, inflammation, and oncogenic insults^17–19^. Based on accumulated evidence, Gdf15 participates in cell homeostatic responses and plays a protective role in both physiological and pathological states, including chronic inflammation and tumorigenesis^20–23^. Given that tissue expression levels of Gdf15 are strongly correlated with secreted levels of Gdf15^24^, circulating Gdf15 levels could potentially represent tissue injuries. Notably, blood Gdf15 levels have been positively associated with renal dysfunction or injury during aging or disease progression, including CKD^24–26^.

In response to diverse internal and external stressors, eukaryotic cells activate a common adaptive pathway, the integrated stress response (ISR), to restore cellular integrity. The core biochemical event in ISR is the phosphorylation of eukaryotic translation initiation factor 2 alpha (eIF2α) by the eIF2α kinase family, which causes global translational arrest and the induction of specific stress-responsive genes to achieve biological homeostasis^27,28^. Herein, we determined whether ISR-driven cellular stress contributes to pathophysiological outcomes via the modulation of Gdf15 during renointestinal distress. Based on the assumption that Gdf15 is a stress-responsive molecule associated with tissue injury, its actions in renointestinal distress were predicted using the clinical transcriptome and animal injury models. In addition to adverse renal outcomes, a mucosa-specific niche, including the microbial community and gut epithelial barrier, was addressed in the complex interorgan communications between the gut and kidney. Our findings would provide novel insights into the prognosis and intervention of renointestinal distress.

## Results

### ISR mediates Gdf15 expression during renal injury

**Fig. 1:**
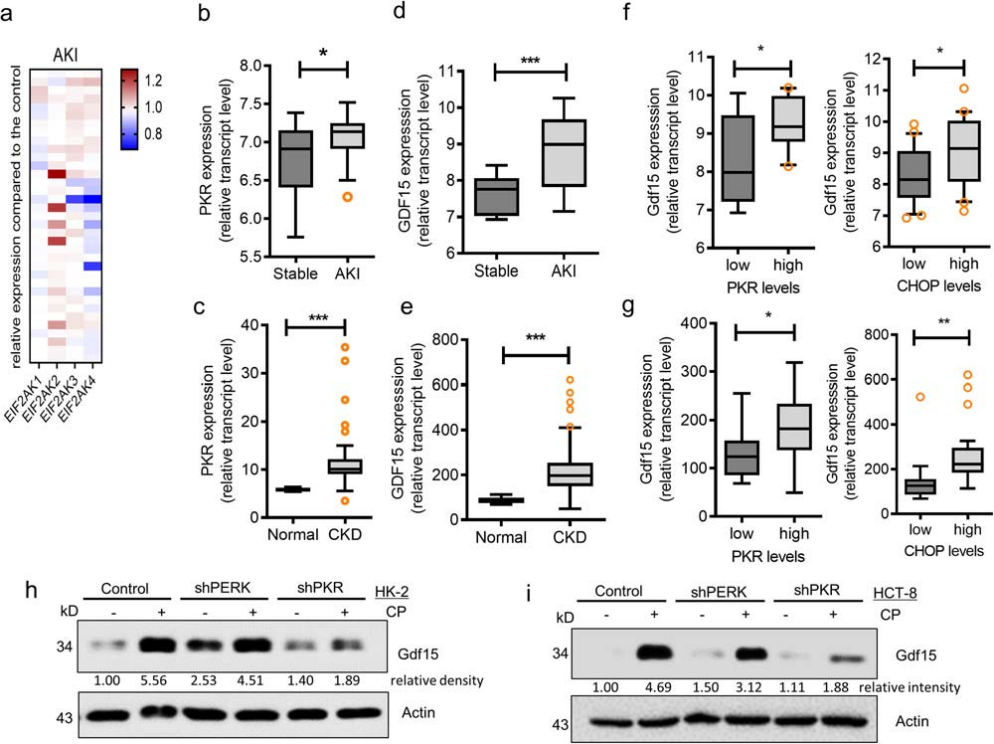
Involvement of integrated stress response (ISR) in renal injuries. **a** Expression levels of eIF2α kinase genes (*EIF2AK1*, *EIF2AK2*, *EIF2AK3*, and *EIF2AK4*) were compared in each patient with acute kidney injury (GSE30718). **b–e** Expression of *PKR* (b, c) or *Gdf15* (**d**, **e**) was determined in patients with renal injuries, including acute kidney injury (GSE30718, b and d) or chronic kidney disease (GSE66494, c and e). The results are shown as a plot with Tukey whiskers. The asterisks (∗) indicate significant differences between the two groups (∗*p* < 0.05, ***p < 0.001). **f**–**g** Based on *PKR* or C/EBP homologous protein (*CHOP*) levels, we selected the 20 highest and 20 lowest level samples, which were further evaluated based on the *Gdf15* levels in patients with renal injuries, including acute kidney injury (GSE30718, **f**) or chronic kidney disease (GSE66494, g). The results are shown as a plot with Tukey whiskers and outliers (orange circles). The asterisks (∗) indicate significant differences between groups (∗*p* < 0.05, ∗∗*p* < 0.01 using a two-tailed unpaired Student’s *t*-test). **h–i** HK-2 cells (**h**) and HCT8 cells (**i**) transfected with control (the negative control shRNA) or shPERK or shPKR plasmid were treated with vehicle or 10 μmol/L cisplatin (CP) for 48 h. Cellular lysates were subjected to western blot analysis. eIF2α, eukaryotic initiation factor 2 alpha; Gdf15, growth differentiation factor 15; PKR, protein kinase R; PERK, protein kinase-like endoplasmic reticulum kinase.

Based on the assumption that ISR-linked cellular processes are involved in modulating renal distress, the expression of four global stress-related mammalian eIF2α kinases, i.e., *EIF2AK1* (*HRI*), *EIF2AK2* (protein kinase R; *PKR*), EIF2AK3 (protein kinase R-like endoplasmic reticulum kinase; *PERK*), and *EIF2AK4* (*GCN2*), were compared in patients with AKI (Fig. 1a). *EIF2AK2* (*PKR*) expression was markedly elevated when compared with the expression levels of other mammalian eIF2α kinases. Clinical transcriptome-based analysis revealed that patients with AKI or CKD exhibited elevated *PKR* levels (Fig. 1b, c). Although *PERK* levels were not significantly elevated in patients with AKI, increased levels were observed in those with CKD (Supplementary figure s1a, b). We also analyzed *Gdf15* expression associated with human AKI and CKD in clinical genomic datasets (GSE30718 and GSE66494, respectively). We observed that the relative expression of *Gdf15* was significantly higher in patients with AKI and CKD than in the control group (Fig. 1d, e, respectively). Moreover, subjects with elevated levels of *PKR* or its representative target, C/EBP homologous protein (*CHOP*), tended to exhibit higher *Gdf15* levels than those with low *PKR* or *CHOP* expression (Fig. 1f [AKI] and 1 g [CKD]), thereby indicating a positive association between *PKR* signaling and *Gdf15* expression. In contrast, *PERK* expression was not significantly associated with *Gdf15* levels in patients with AKI or CKD (Supplementary figure s1c, d, respectively).

Gdf15 was mechanistically evaluated as a stress-induced factor in response to platinum exposure during renointestinal distress. Consistent with the clinical transcriptomic evaluation, the renal stress response to CP was assessed using HK-2 cells, a well-established immortalized proximal tubule epithelial cell line derived from normal adult human kidneys^29^. Furthermore, intestinal stress responses have been simulated in the HCT-8 cell line, a widely employed human intestinal epithelial cell model for inflammatory and infectious diseases^30,31^. Based on the 24-h cellular viability (Supplementary Fig. s2a, b), the following cell-based evaluations were performed at CP doses corresponding to IC_50_ values at each given exposure time. In response to CP treatment, HK-2 and HCT-8 cells showed increased Gdf15 expression (Fig. 1h, i). In addition to genotoxic stress, CP has been found to disrupt organelle functions, including translational machinery, via strong binding to RNA and ribosomes^32–37^. Therefore, among the various types of eIF2α kinase-linked signaling pathways in ISR, RNA-dependent PERK- and double-stranded RNA-dependent PKR-associated signals were examined to determine their possible impact on Gdf15 expression levels. Specific small hairpin RNAs (shRNAs) were used to knock down PERK or PKR to determine their impact on Gdf15 expression. Compared with PERK expression, PKR was markedly involved in CP-induced Gdf15 expression in both cell types. These findings confirmed that PKR-linked signaling is critically involved in Gdf15 expression during renal injury.

### Gdf15 deficiency aggravates acute renal and mucosal injuries

**Fig. 2:**
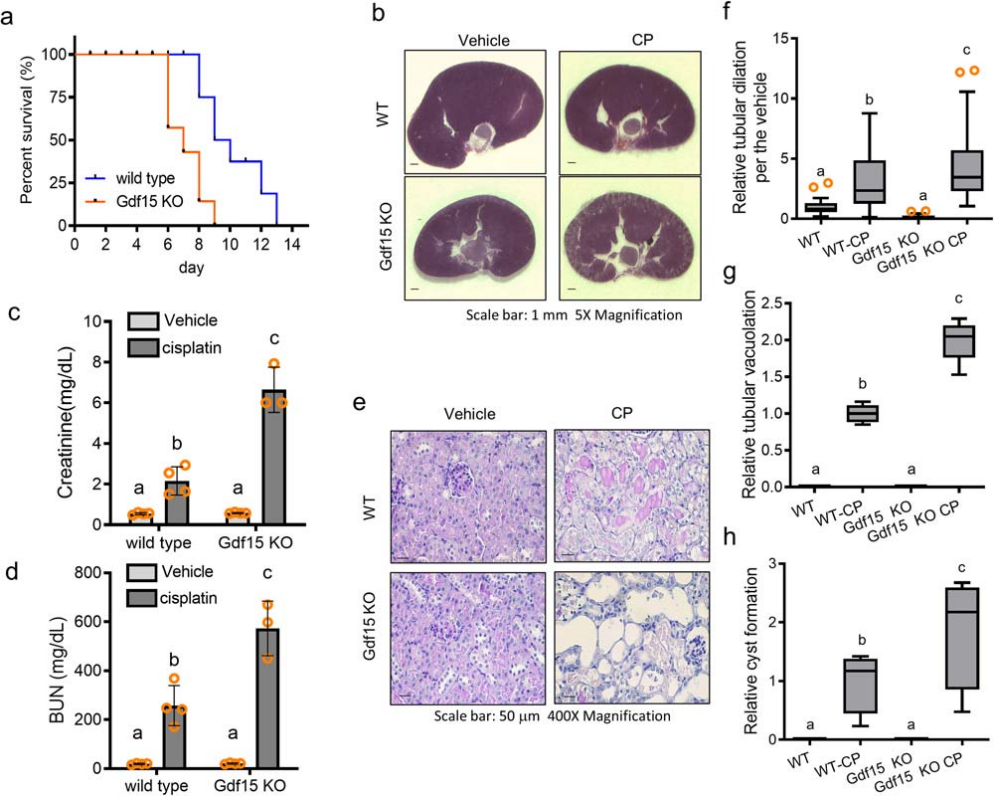
Role of *Gdf15* in cisplatin (CP)-induced renal injuries. Eight-week-old wild-type and Gdf15 knockout (KO) mice were treated with vehicle or CP (20 mg/kg, intraperitoneal) for 72 h (*n* = 3 − 5). **a** Kaplan-Meier’s survival analysis of wild-type and Gdf15 KO mice treated with CP (*n* = 3 − 5, *p* < 0.01). **b** Gross anatomy of kidney sections from untreated mice and CP-treated mice (periodic acid-Schiff [PAS] staining) (Magnification, 5×; Scale bars(s), 1 mm). Serum levels of creatinine (**c**) and blood urea nitrogen (BUN) (**d**) were measured at 72 h after CP treatment using a colorimetric assay kit, as described in the method section. The results are shown as a bar graph with average and standard deviation, and different letters over each bar represent significant differences between groups (*p* < 0.05). **e** Histological examination of PAS-stained kidney sections (Magnification, 400×; Scale bars(s), 50 μm). Quantitative analysis of tubular dilation (**f**), tubular vacuolation (**g**), and cyst formation (**h**). The results are shown as a plot with Tukey whiskers and outliers (orange circles). Different letters over each bar represent significant differences between groups (*p* < 0.05). Gdf15, growth differentiation factor 15.

Specifically, we evaluated whether high levels of Gdf15 expression were related to the prognosis of renal pathology in a mouse model of CP-induced AKI. The Kaplan-Meier survival plot revealed that Gdf15 deficient mice displayed a poorer prognosis than wild-type mice following CP treatment (Fig. 2a); this indicated the protective actions mediated by Gdf15 against acute renal injury and adverse outcomes. Gross anatomical observation of the kidneys revealed extensive tissue damage in Gdf15 knockout (KO) mice in response to CP treatment when compared with that in wild-type mice (Fig. 2b). Creatinine and blood urea nitrogen (BUN) levels were measured to verify renal functional injuries. Following CP treatment, creatine and BUN levels were significantly higher in Gdf15 KO than in wild-type mice (Fig. 2c, d, respectively). Furthermore, we performed a quantitative evaluation of renal tissue injuries using periodic acid-Schiff (PAS) staining (Fig. 2e) and found a high degree of tubular dilation and tubular vacuolation in Gdf15 KO mice when compared with that in wild-type mice in response to CP (Fig. 2f, g). In addition, CP-induced cyst formation was notable in Gdf15 KO mice when compared with that in wild-type mice (Fig. 2h). Overall, these results indicated a protective role for Gdf15 against CP-induced renal injury.

**Fig. 3:**
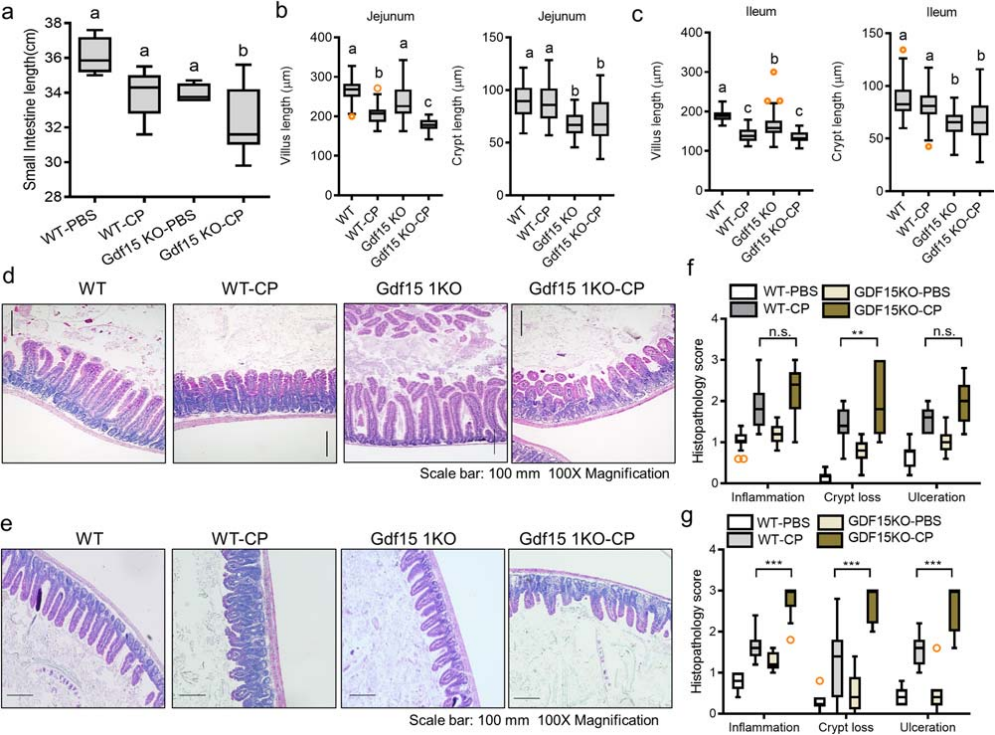
Effects of Gdf15 deficiency on gut morphology during renal injury. Eight-week-old wild-type and Gdf15 knockout (KO) mice (*n* = 3–5) were treated with vehicle or cisplatin (CP; 20 mg/kg) for 72 h. **a** The average length of the small intestine from each group. **b–c** Average lengths of villus (left) and crypt (right) in the jejunum (**b**) and ileum (**c**). The results are shown as a plot with Tukey whiskers and outliers (orange circles). Different letters over bars represent significant differences between groups (*p* < 0.05). **d–g** Histological examination of hematoxylin and eosin (H&E)-stained sections of the jejunum (**d**) and ileum (**e**) (Magnification, 100×; Scale bar(s), 100 μm). Quantitation of pathological severity of the jejunum (**f**) and ileum (**g**). The results are shown as a plot with Tukey whiskers and outliers (orange circles). The asterisks (∗) indicate significant differences between groups (∗∗*p* < 0.01, ∗∗∗*p* < 0.001 using a two-tailed unpaired Student’s *t*-test). Gdf15, growth differentia*t*ion factor 15.

In addition to renal distress, mucositis is a known complication associated with CP-induced complication^14^. We examined the effects of Gdf15 deficiency on CP-induced mucositis in mice. Based on gross anatomical observations, Gdf15 deficiency significantly aggravated CP-induced shortening of the mouse small intestine considering the longitudinal length (Fig. 3a). Following microscopic examination of the intestinal epithelial layer, we detected blunting or shortening of the villi or crypts in CP-exposed mice, which was markedly severe in Gdf15 KO mice (Fig. 3b, c). Gdf15-associated shortening of the lining length, villi, and crypts suggested a reduction in the small intestinal luminal surface available for nutrient absorption. Furthermore, histopathological scoring of pathological severity revealed that Gdf15 deficiency could aggravate CP-induced crypt loss (Fig. 3d–g). Notably, Gdf15-deficiency enhanced inflammation and ulceration, which were more pronounced in the ileum than in the jejunum (Fig. 3f, g).

**Fig. 4:**
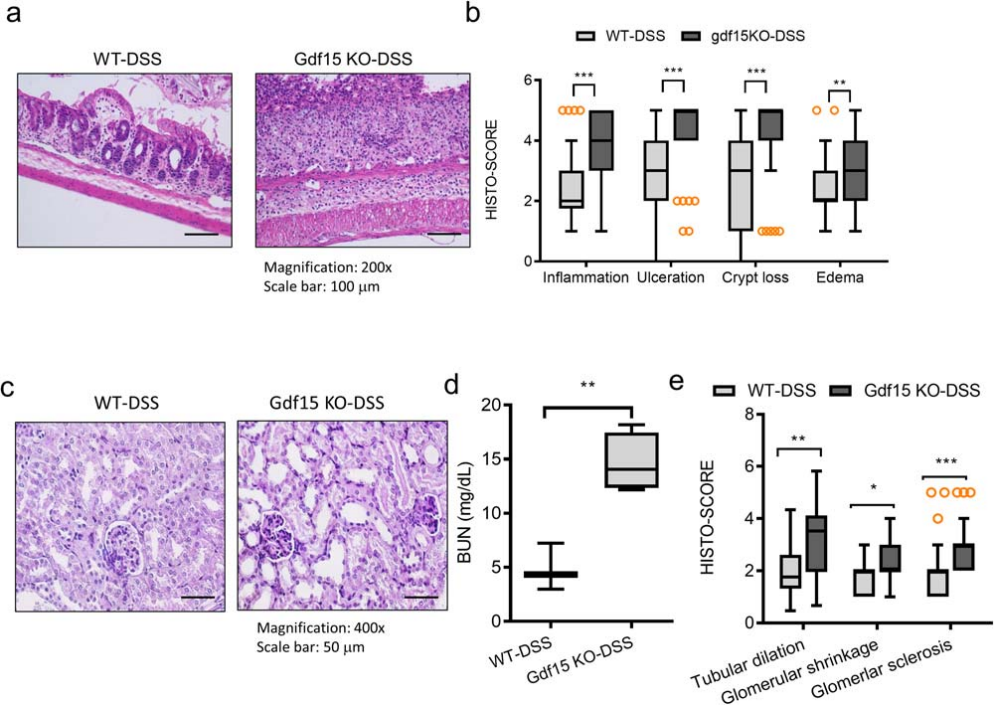
Effects of Gdf15 deficiency on gut pathology in dextran sodium sulfate (DSS)-exposed mice. Eight-week-old wild-type and Gdf15 knockout (KO) mice (*n* = 3-5) were treated with vehicle or 3% DSS for 8 days. Histological examination of hematoxylin and eosin (H&E)-stained colon sections (**a**). Quantitation of pathological severity of colons (**b**) (Magnification, 200×; Scale bar(s), 100 μm). The results are shown as a plot with Tukey whiskers and outliers (orange circles). The asterisks (∗) indicate significant differences between groups (∗∗*p* < 0.01, ∗∗∗*p* < 0.001). **c** Histological examination of periodic acid-Schiff (PAS)-stained kidney sections (Magnification, 400×; Scale bars(s), 50 μm). **d** Blood urea nitrogen (BUN) was measured 48 h after DSS treatment using a colorimetric assay kit. The results are shown as a plot with Tukey whiskers. The asterisks (∗) indicate significant differences between groups (**p < 0.01). **e** Quantitative analysis of tubular dilation, glomerular shrinkage, and glomerular sclerosis. The results are shown as a plot with Tukey whiskers and outliers (orange circles). The asterisks (∗) indicate significant differences between the two groups (*p < 0.05, **p < 0.01, ***p < 0.001). Gdf15, growth differentiation factor 15.

In addition to CP-induced intestinal injuries, we assessed a well-defined ulcerative colitis model using dextran sodium sulfate (DSS). In response to DSS treatment, Gdf15 KO mice exhibited increased levels of inflammation, ulceration, crypt loss, and edema in the colon when compared with those in wild-type mice (Fig. 4a, b). We assessed a representative model of chronic kidney disease via treatment with a high-fat diet (HFD) (Supplementary Figure s3). In this model, we could not find notable histological defects in the gut while chronic exposure to HFD (12 weeks) caused renal injuries such as tubular dilation. However, Gdf15 did not contribute to HFD-induced renal pathological events. On an assumption that gut injury is associated with adverse outcomes in the kidney, we further determined whether colitis-inducing insult affects renal integrity. Animals with the dextran sodium sulfate (DSS)-induced ulcerative colitis displayed pathological outcomes in the kidney, including elevated BUN levels and tubular dilation, which were notable Gdf15 KO animals (Fig. 4c, d). In addition to tubular distress, we found that Gdf15 deficiency increased glomerular injuries, including morphological shrinkage and neutrophil infiltration in the DSS-induced colitis model (Fig. 4e). Collectively, Gdf15 deficiency contributed to histological alterations and pathological severity in the renointestinal injury models.

### Gdf15 is involved in counteracting microbiota-associated mucosal barrier degradation

**Fig. 5:**
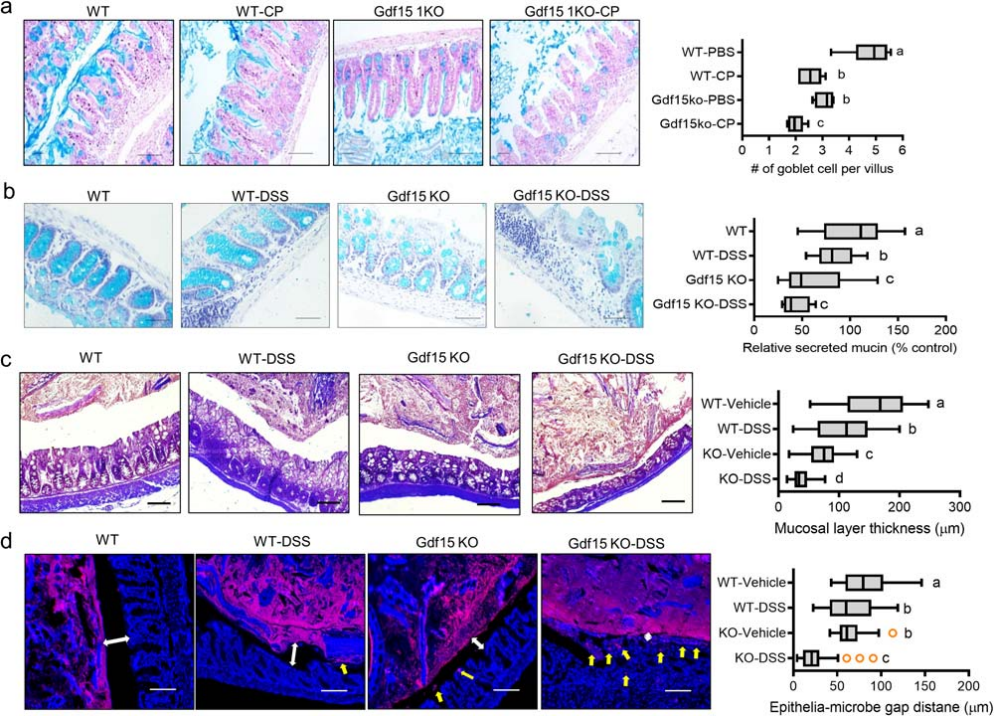
Effects of Gdf15 deficiency on gut mucosa during renal injury. **a** Eight-week-old wild-type and Gdf15 knockout (KO) mice (*n* = 3-5) were treated with vehicle or cisplatin (CP; 20 mg/kg) for 72 h. Staining of ileum mucosa with Alcian blue (Magnification, 200×; Scale bar(s), 100 μm) and its quantitation (Magnification, 200×; Scale bar(s), 100 μm). **b–d** Eight-week-old wild-type and Gdf15 KO mice (*n* = 3–5) were treated with vehicle or 3% DSS for 8 days. Staining of ileum mucosa with Alcian blue (Magnification, 200×; Scale bar(s), 100 μm) and its quantitation (Magnification, 200×; Scale bar(s), 100 μm) (**b**). Representative images of the intestinal mucosal bacteria using Gram staining (**c**) or 16 rRNA in situ staining (**d**) (Magnification, 200×; Scale bar(s), 100 μm). The mucosal layer thickness was measured (the right graph). The quantitation analysis was shown as plots with Tukey whiskers and outliers (orange circles). Different letters with bars represent significant differences between groups (*p* < 0.05). Gdf15, growth differentiation factor 15.

The gut mucosal layer was further evaluated, given that it is a pivotal barrier against proinflammatory triggers, including microbes and their components. Considering mucosal defense, CP exposure reduced the number of goblet cells and decreased mucus secretion in the ileal epithelial layer, which was further exacerbated in Gdf15 KO mice (Fig. 5a, b). In addition to the small intestine, Gdf15 deficiency depleted colonic mucin secretion, thereby indicating the protective function of Gdf15 for goblet cells or their mucin production. Therefore, Gdf15 KO also induced severe loss of the mucosal layer in the DSS-induced colitis model (Fig. 5c). Gram staining was performed to localize the luminal bacteria and estimate the thickness of the inner mucosal layer. Gdf15-deficient epithelial cells were in close contact with the luminal matter containing dietary and microbial factors, given the reduced mucosal barrier (Fig. 5d). Subsequently, luminal bacteria could easily translocate into the inner tissues and play crucial roles in triggering inflammatory insults in the vasculature and other target organs, including the kidneys. The present evidence suggests that Gdf15-mediated integrity of the epithelial and mucosal barrier counteracts the circulatory release of gut-derived renotoxic or proinflammatory factors.

**Fig. 6:**
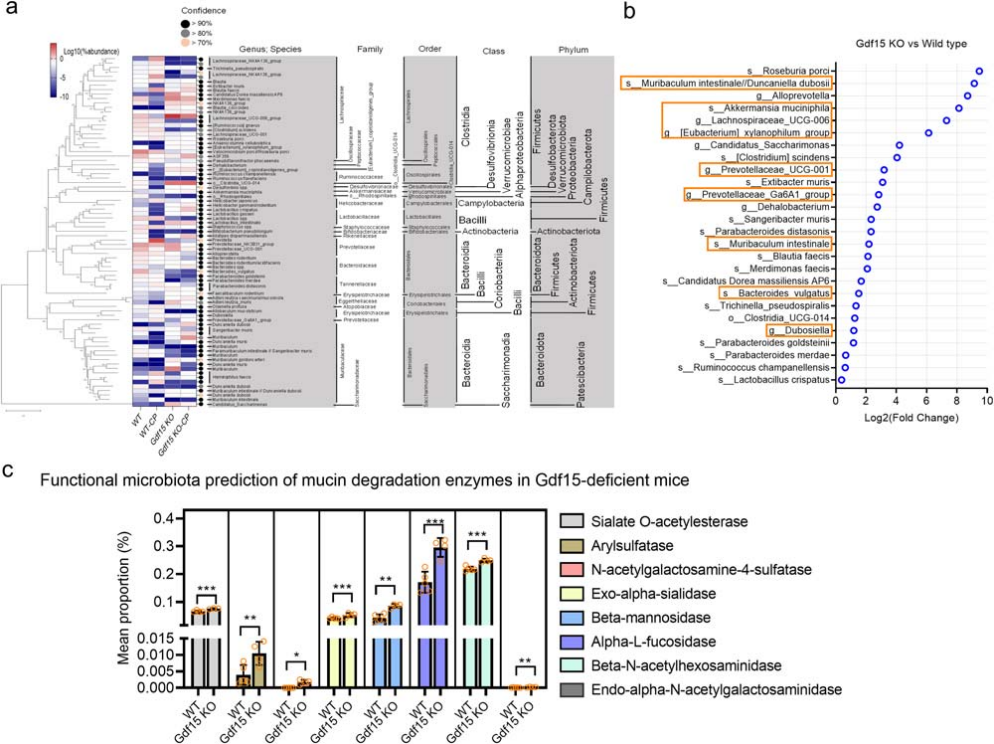
Effects of Gdf15 deficiency on gut microbiota. The fecal bacteria were subjected to 16 S rRNA analysis to determine the phylogenetic composition. **a,b** The bacteria of each top 30 abundant taxa are listed along with the corresponding abundance (**a**) and the relative abundance (**b**). Re-marked microbes are potent mucin-foraging genera or species. **c** Genes related to mucin degradation enzymes reconstructed from 16 S rRNA profile with PICRUSt2. Data represent the mean ± standard deviation (SD) (*N* = 3) with all datapoints (circles) (results are shown as the mean values ± SD. Asterisks (∗) indicate significant differences between groups (∗*p* < 0.05, ∗∗*p* < 0.01, ∗∗∗*p* < 0.001). Gdf15, growth differentiation factor 15.

In addition to the altered host tissue barrier, we evaluated the luminal bacterial composition as a potential etiology associated with the aggravated gut barrier, as mucosal microbes can modulate the synthesis or degradation of the mucosal matrix. In total, 1,821,852 paired-end reads were produced from the three libraries using the Illumina iSeq platform (Illumina Inc., San Diego, CA, USA). Subsequently, the 1,821,852 single-end reads were subjected to further analyses using the QIIME2 (ver.2021.2) pipeline. Using the DADA2 algorithm, 2,012 representative sequences of the V4 region of 16 S rRNA genes, with an average length of 151 bp across three libraries, were constructed, among which 100047.6, 102951.5, 100358.5, and 114681.25 average features were counted in wild-type (vehicle), Gdf15 KO (vehicle), wild-type (CDDP), and Gdf15 KO (CDDP), respectively. Alpha diversity based on the Shannon, Simpson, and Chao1 metrics revealed that the degree of diversity within each microbial community was similar (Supplementary figure s4a). In contrast, beta diversity significantly differed between communities (Supplementary figure s4b). Moreover, the composition analysis of the phyla demonstrated that Firmicutes and Bacteroidota were the most dominant phyla in all samples (Supplementary figure s4c). The composition differed across samples, as Firmicutes contributed 56.9 and 59.6% of the total features as the most abundant phylum in the vehicle and CDDP groups, respectively, whereas the relative abundance of Bacteroidota was increased in Gdf15 KO mice (Supplementary figure s4a). Additionally, the ratio of Proteobacteria was 9-fold its original value in response to CDDP exposure in wild-type mice, whereas the CDDP-induced elevation in Proteobacteria abundance was marginal in Gdf15 KO animals (Supplementary figure s4c). An in-depth assessment of the communities at the family or genus level demonstrated that unclassified Lachnospireae, as the major family belonging to the phylum Firmicutes, contributed 25–32% in each group (Fig. 6a). However, *Muribaculaceae* was the main genus in the phylum Bacteroidota in the Gdf15 KO animals. Further evaluation was performed to rank the relative abundance of bacteria at the species level in response to Gdf15 deficiency. In particular, Gdf15 deficiency increased the relative abundance of *Muribaculum intestinale, Duncaniella dubosii, Akkermansia muciniphila, Lachnospiraceae_UCG-006, Prevotellaceae, Bacteroidesvulgatus*, and *Dubosiella* (Fig. 6b and Supplementary figure s4d), all of which were identified as mucin glycan foragers in the human gut microbiome^38,39^. Furthermore, metagenomic prediction based on the profile using *PICRUSt2* (ver.2.3.0) demonstrated significant elevations in genes encoding mucin-degradation-related enzymes in response to Gdf15 deficiency (Fig. 6c). In particular, Gdf15 deficiency significantly enhanced expression levels of genes for sialate O-acetylesterase (EC:3.1.1.53), arylsulfatase N-acetylgalactosamine-4-sulfatase (EC:3.1.6.1), exo-alpha-sialidase (EC:3.1.6.12), beta-mannosidase (EC:3.2.1.18), alpha-l-fucosidase (EC:3.2.1.25), beta-N-acetylhexosaminidase (EC:3.2.1.51), and endo-alpha-N-acetylgalactosaminidase (EC:3.2.1.52). Taken together, Gdf15 deficiency concomitantly increased the abundance of mucin metabolism-linked bacteria and enzymes along with mucosal barrier disruption, indicating that Gdf15 counteracts mucin degradation induced by microbes.

### Gdf15-linked autophagy signaling mediates mucin production and cellular protection

**Fig. 7:**
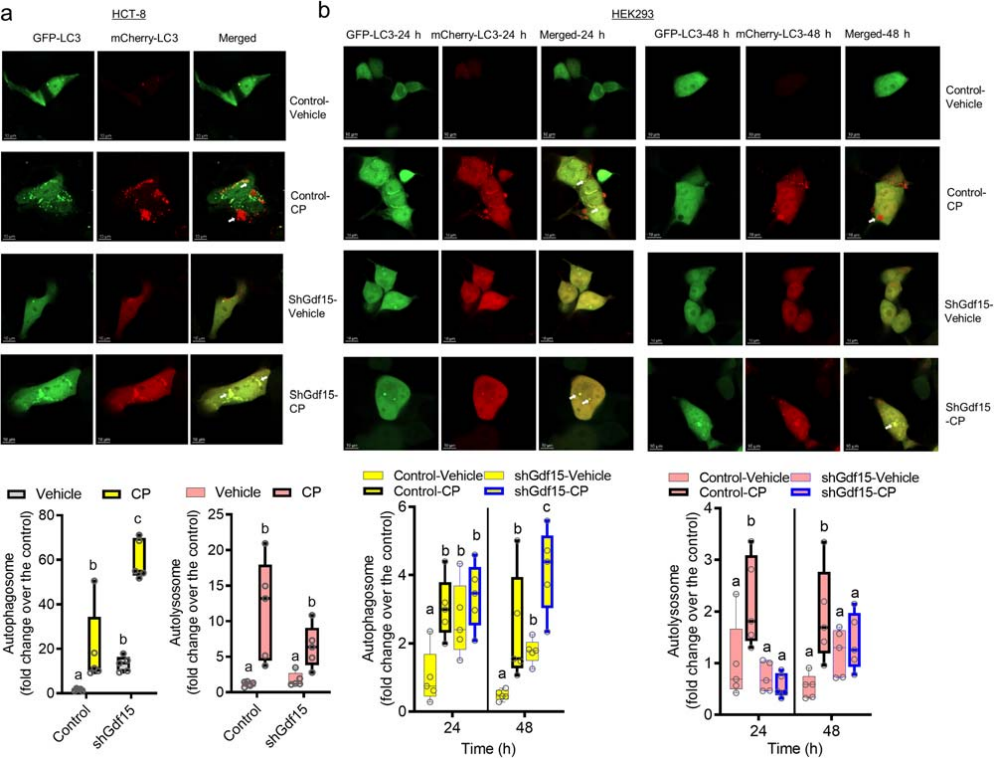
Effects of Gdf15 on autophagy in cells. Control or Gdf15-deficient cells (HCT-8 (**a**) or HK-2 (**b**) cells) were transfected with pDEST-CMV mCherry-GFP-LC3B WT. At 24 h after transfection, cells were treated with vehicle or 10 μmol/L cisplatin (CP) for 48 h. Cells were observed under the confocal microscope to monitor the pH-responsive autophagic flux (Magnification, 400×; Scale bar(s), 10 μm). The quantitation analysis was shown as plots with Tukey whiskers (lower graphs) and all datapoints (circles). Different letters with bars represent significant differences between groups (*p* < 0.05). Gdf15, growth differentiation factor 15.

Considering the Gdf15-mediated counteraction against renointestinal injuries, autophagy was evaluated as one of the representative protection mechanisms. First, we assessed the involvement of Gdf15 in the autophagic flux in the gut and kidney cells using the plasmid for expression of mCherry- and GFP-tagged microtubule-associated protein 1 A/1B light chain 3 B (LC3B), a representative autophagosome marker (Fig. 7a, b). The fusion of autophagosomes to late endosomes or lysosomes results in acidic autolysosomes where the signal of acid-sensitive GFP is lost. Moreover, the signal of acid-insensitive mCherry is ultimately lost when the double-tagged protein is degraded. CP-insulted cells exhibited autophagosome formation (the yellow puncta), which was followed by autolysosome formation (the red) for clearance of the ubiquitinylated proteins under chemical stress. However, Gdf15-deficient cells showed defects in the normal autophagic flux. The autolysosome formation was remarkably interfered in Gdf15 knockdown, resulting in the prolonged yellow puncta signal with attenuated levels of red fluorescence puncta (Fig. 7a, b), suggesting the pivotal roles of Gdf15 in facilitating the autophagic flux.

**Fig. 8:**
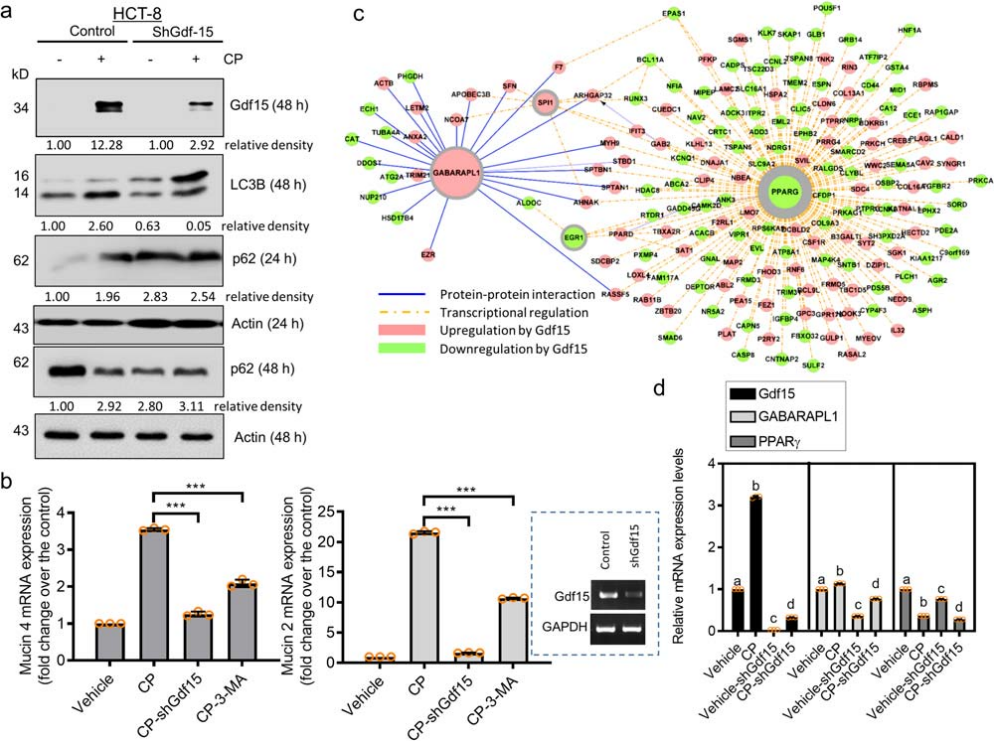
Effects of Gdf15 deficiency on gut autophagy signaling and mucin production. **a** HCT8 cells transfected with control or shGdf15 vector were treated with vehicle or 10 μmol/L cisplatin (CP) for 48 h. Cellular lysates were subjected to western blot analysis. **b** HCT-8 cells transfected with control (the negative control shRNA) or shGdf15 plasmid were treated with vehicle or 10 μmol/L CP in the absence or presence of 20 μmol/L 3-methyladenine (3-MA) for 48 h, and mRNA levels of *mucin 4* or *mucin 2* were measured using reverse transcription-quantitative polymerase chain reaction (RT-qPCR) analysis. Data values are presented as the mean ± standard deviation (SD) and all datapoints (circles). Asterisks (∗) indicate significant differences from the CP-treated group (∗∗∗*p* < 0.001). Boxed blots indicate the efficient suppression of Gdf15 using shRNA. **c** Cluster evaluation of autophagy regulatory network (ARN; https://autophagyregulation.org) in response to Gdf15 levels in human cells. The border thickness indicates the levels of betweenness centrality of each node. **d** The HCT-8 cells transfected with control (the negative control shRNA) or shGdf15 plasmid were treated with vehicle or 20 μmol/L CP for 12 h, and mRNA levels were measured using RT-qPCR analysis. Data values are presented as the mean ± SD and all datapoints (circles). Different letters over bars represent significant differences between groups (*p* < 0.05). Gdf15, growth differentiation factor 15.

Next, we investigated the mechanistic link between Gdf15 and mucosal protection. Accordingly, autophagy was assessed as one of the protective signaling mechanisms against chemical-induced mucositis. We noted that Gdf15-knockdown gut cells exhibited reduced activation of LC3B (Fig. 8a), thereby indicating that Gdf15 positively regulates autophagy. Moreover, we observed p62, a receptor for cargo destined to be degraded by autophagy, which binds to ubiquitin and LC3, facilitating clearance of ubiquitinated proteins in the autolysosome. Although p62 was ultimately degraded in CP-exposed intestinal cells, Gdf15-deficient cells exhibited resistance to the CP-induced degradation process. Considering the mucosa-mediated gut protection, the in vitro evaluation using human intestinal epithelial cells demonstrated Gdf15-dependent expression of *mucins 2* and *4* (Fig. 8b). Moreover, inhibition of autophagy signaling attenuated *mucin 2/4* induction in response to CP-induced distress, indicating that Gdf15-mediated autophagy signaling participates in intestinal mucin expression.

To address the detail signaling network involved in Gdf15-induced autophagy, we performed a systematic bioinformatics analysis of Gdf15 expression in human cells using the autophagy regulatory network (ARN) database, which encompasses various evidence-based autophagy machinery-linked components, their regulators, transcription factors, miRNA regulators, and signaling network connectors in multi-layered illustration^40^. Based on the human Gdf15-responsive gene profile in gut cells, we identified two clusters of receptor-associated autophagy signaling (Fig. 8c). We found that Gdf15 enhanced gamma-aminobutyric acid receptor-associated protein-like 1 (*GABARAPL1)* in the signaling hierarchy system, which mediates signal transduction through protein-protein interactions and downregulates the nuclear receptor peroxisome proliferator-activated receptor gamma (PPAR-γ)-regulated transcriptional network. GABARAPL1 and PPAR-γ are key components with high betweenness centrality of ARN in response to Gdf15 levels. Moreover, GABARAPL1-modulated high-degree transcription factors, such as Sp1 and early growth response gene 1 (Egr1), contributed to Gdf15-responsive network alterations. Additionally, we examined Gdf15-regulated critical modules of the autophagy network in CP-exposed human intestinal cells. Gut epithelial exposure to CP slightly enhanced *GABARAPL1* expression, which was positively regulated by Gdf15 (Fig. 8d). In contrast, chemical stress downregulated the expression of *PPARγ*.

**Fig. 9:**
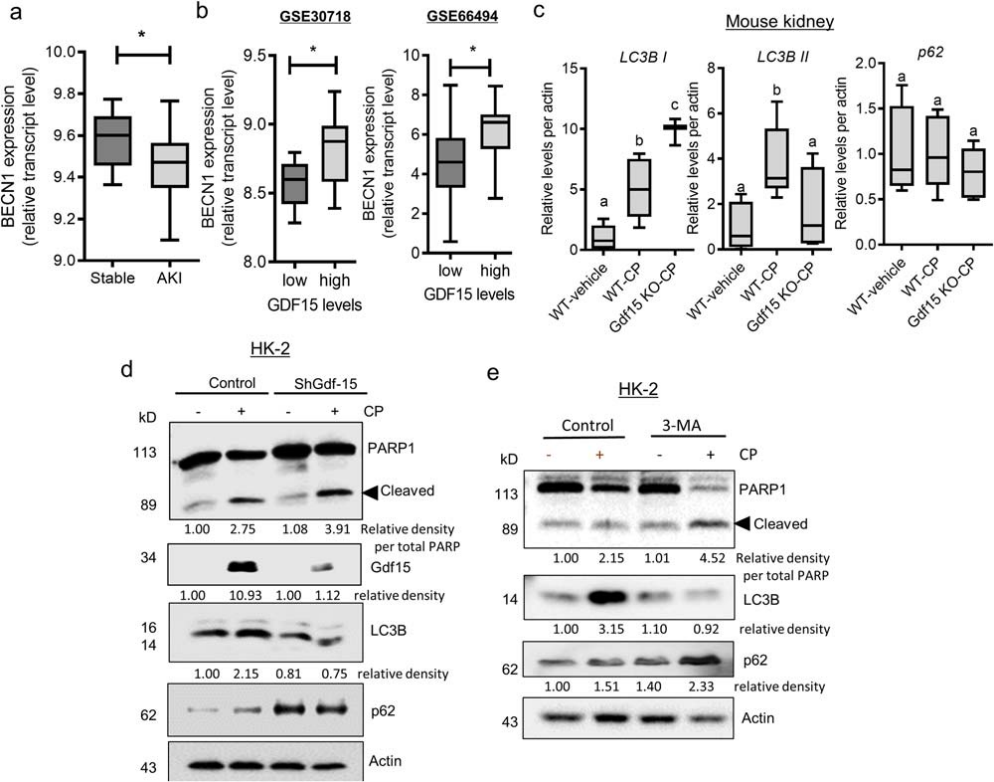
Effects of Gdf15 and autophagy on intestinal mucin production. **a** Expression of *BECN1* in patients with acute kidney injury (GSE30718). **b** Based on the *Gdf15* levels in patients with acute kidney injury (GSE30718, left) or chronic kidney disease (GSE66494, right), we selected the 20 highest and 20 lowest level samples, followed by a comparison of *BECN1* expression levels. The results are shown as a plot with Tukey whiskers. The asterisks (∗) indicate significant differences from the low-expression group (∗*p* < 0.05 using a two-tailed unpaired Student’s *t*-test). **c** Eight-week-old wild-type and Gdf15 knockout (KO) mice (*n* = 3–5) were treated with vehicle or cisplatin (CP; 20 mg/kg) for 72 h. Kidney tissue lysates were subjected to western blotting. The results are shown as a plot with Tukey whiskers. The graph shows the relative densities of light chain 3 B (LC3B) from the western blot. Different letters over bars represent significant differences between groups (*p* < 0.05, *n* = 3). **d** HK-2 cells transfected with control or shGdf15 vector were treated with vehicle or 10 μmol/L CP for 48 h. Cellular lysates were subjected to western blot analysis. **e** HK-2 cells were treated with vehicle or 10 μmol/L CP in the absence or presence of 20 μmol/L 3-methyladenine (3-MA) for 48 h. Cellular lysates were subjected to western blot analysis. Gdf15, growth differentiation factor 15.

Next, we aimed to predict the link between autophagy and renal tissue injury. Although the precise role of autophagy in kidney disease remains controversial, patients with AKI tend to exhibit reduced levels of Beclin-1 (*BEC1*), a representative autophagy biomarker (Fig. 9a). Clinical transcriptome analysis also revealed that subjects with high *Gdf15* expression tended to exhibit increased levels of *BEC1* during acute or chronic renal injury (Fig. 9b). Experimentally, *Gdf15* KO mice exhibited reduced expression of LC3B II, a representative autophagosome marker (Fig. 9c), thereby indicating that Gdf15 positively regulates autophagy. Consistent with renal tissue expression levels, Gdf15 knockdown in HK2 cells attenuated the activation level of LC3B (Fig. 9d). Moreover, Gdf15-deficient cells displayed enhanced levels of p62, a receptor for cargo destined to be degraded by autophagy, indicating that Gdf15 contributes to the autolysosome process as well as the autophagosome formation in the renal tubular cells. In contrast, Gdf15 suppression increased CP-induced renal cell death (PARP1/2 cleavage), indicating the protective effects of Gdf15 via autophagy signaling in response to CP exposure. Furthermore, the inhibition of autophagy signaling aggravated CP-induced renal cell death (Fig. 9e). Collectively, Gdf15 and its downstream autophagy pathway mechanistically afford protection against renal cell death.

**Fig. 10:**
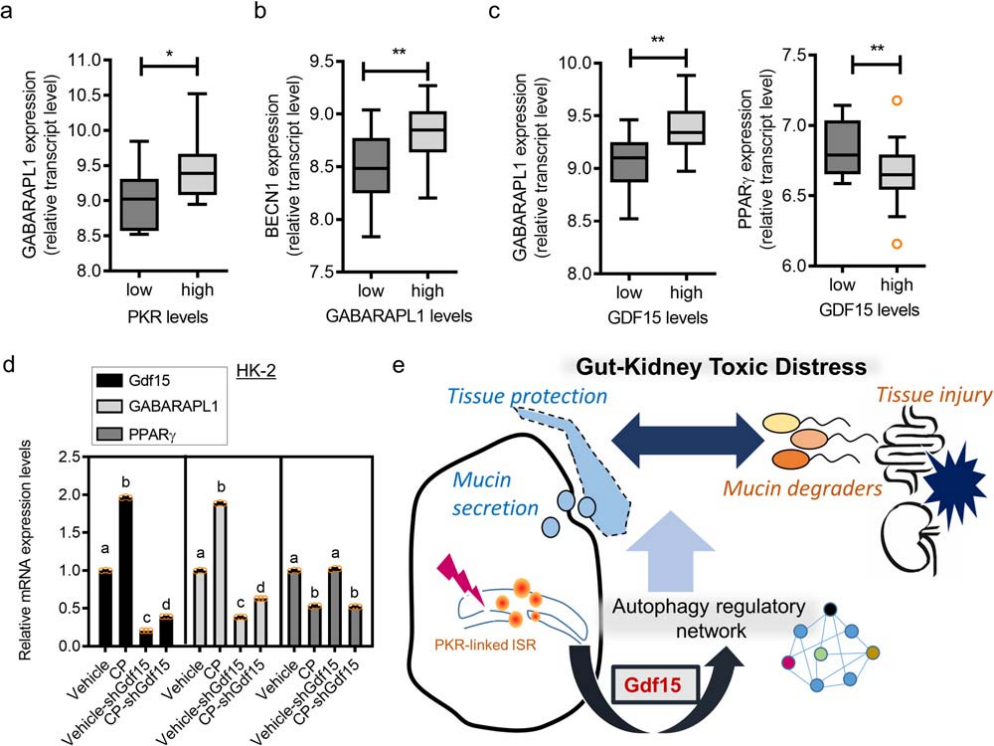
Clinical transcriptome analysis of autophagy regulatory modules in response to Gdf15. **a** Based on protein kinase R (*PKR*) levels in patients with acute kidney injury (GSE30718), we selected the 20 highest and 20 lowest samples, followed by a comparison of gamma-aminobutyric acid receptor-associated protein-like 1 (*GABARAPL1*). **b** Based on the *GABARAPL1* levels in patients with acute kidney injury (GSE30718), we selected the 20 highest and 20 lowest samples, followed by a comparison of *BECN1* expression. **c** Based on the *Gdf15* levels, we selected the 20 highest and 20 lowest samples, followed by a comparison of *GABARAPL1* (left) and *PPAR-γ* (right) expression in patients with acute kidney injury (GSE30718). The results are shown as a plot with Tukey whiskers and outliers (orange circles). The asterisks (∗) indicate significant differences from the low-expression group (*p < 0.05, **p < 0.01 using a two-tailed unpaired Student’s *t*-test). **d** The HK-2 cells transfected with control (the negative control shRNA) or shGdf15 plasmid were treated with vehicle or 20 μmol/L cisplatin (CP) for 12 h, and mRNA levels were measured using reverse transcription-quantitative polymerase chain reaction analysis. Data values are presented as the mean ± standard deviation (SD), and different letters over bars represent significant differences between groups (*p* < 0.05). **e** Mechanistic scheme of Gdf15-linked protection. Cells activate integrated stress responses following tubular or mucosal insults, leading to Gdf15 production. Stress-responsive Gdf15 plays crucial roles in reorganizing the autophagy regulatory network for endogenous adaptation, including intestinal mucin production and renal cell survival against cytotoxic injuries. Moreover, the protection-linked Gdf15 counteracted the mucus-degrading microbiota in the distressed gut. Gdf15, Growth differentiation factor 15; PPAR-γ, peroxisome proliferator-activated receptor gamma.

Finally, clinical transcriptome analysis verified the autophagy signaling in patients with acute renal distress. Considering the integrated stress responses during renal injury, PKR eIF2α kinase-mediated stress signaling was critically involved in Gdf15 induction during renal injury and CP exposure (Fig. 1). Accordingly, we evaluated *GABARAPL1* expression and *PKR* levels in patients with AKI. Subjects with elevated levels of *PKR* exhibited increased *GABARAPL1* expression, indicating that ISR enhances *GABARAPL1* expression (Fig. 10a). Furthermore, PKR-responsive *GABARAPL1* was positively associated with improved levels of *BECN1*, an autophagy biomarker in injured renal tissues (Fig. 10b). Moreover, patients with high *Gdf15* levels tended to demonstrate increased *GABARAPL1* levels and decreased *PPARγ* expression, suggesting a Gdf15-altered autophagy network during acute renal distress (Fig. 10c). Furthermore, the clinical database-linked prediction of the regulatory patterns of two key autophagy network molecules, *GABARAPL1* and *PPAR-γ* was similar to those observed in the CP-exposed renal tubular cells (Fig. 10d). Collectively, stress-responsive Gdf15 modulated the autophagy signaling network, contributing to the maintenance of mucosal integrity and renal cell survival in the distressed gut-kidney axis (Fig. 10e).

## Discussion

In the present study, Gdf15 was evaluated as an endogenous adaptation molecule against pathological stress. Notably, ISR-activated Gdf15 facilitated cellular protection via signaling reprogramming of the autophagy network in response to tissue injury. In addition to renal cell protection, the Gdf15-linked autophagic signaling was involved in gut barrier maintenance via regulation of mucin production against mucus-degrading microbiota during the renal injury. Clinical dataset- and experiment-based predictions and evidence on Gdf15-mediated protection provide insights into robust targets for molecular prevention or intervention against intestinal and renal injury. Gdf15 has also been assessed as an early biomarker of various tissue injuries, regulating inflammation, cell survival, proliferation, and apoptosis during disease progression^41^. Moreover, increased serum levels of Gdf15 have been associated with an enhanced risk of CKD progression, particularly indicating its usefulness as a marker of renal impairment in the elderly and young populations^25,42^. Moreover, urinary Gdf15 levels can be associated with adverse outcomes, kidney histology patterns, and the need for renal replacement therapy in patients with CKD^43^. Kidney transplantation reportedly reduces serum Gdf15 levels, whereas nephrectomy was found to upregulate these levels, indicating that renal Gdf15 regulates various physiological functions^44^. Herein, ISR-linked Gdf15 could be a valuable prognostic biomarker for acute renointestinal distress, but additional evaluations are warranted to generalize the prediction in diverse other renal injuries

Gdf15 facilitates the reorganization of ARN in response to cellular stress. However, mechanistic evidence regarding Gdf15-mediated regulation of autophagy remains limited. In terms of indirect evidence of Gdf15 action in autophagy, Gdf15 combined with oxidized low-density lipoprotein was shown to impair autophagic processes by regulating the expression of autophagy-relevant proteins in human macrophages during atherosclerotic plaque formation^45^. In the autophagy machinery, GABARAPL1 was predicted to be a protective signaling mediator in PKR-linked renal pathogenesis following Gdf15 induction. In mammalian cells, cargo membranes are recognized and conjugated by the autophagy-related protein 8 family, including GABARAPs or LC3 proteins, which play critical roles in autophagic flux^46,47^. Although LC3 is involved in elongating the phagophore membrane, GABARAPs, including GABARAPL1, are essential for phagophore extension and sealing, resulting in autophagosome maturation^48^. Moreover, the GABARAP subfamily positively regulates ULK1 (Unc-like autophagy activating kinase1), a key stress-responsive kinase that initiates the autophagy machinery in response to internal or external stressors, including nutrient and metabolic stress signaling^49^. In the present study, stress-responsive PKR and Gdf15 expression were positively associated with GABARAPL1 expression in patients with acute renal injury, which could facilitate early autophagy formation and maturation as a protective cell process against renal tubular and intestinal epithelial injuries. Although the role of autophagy in kidney injury needs to be comprehensively elucidated, early stress-responsive autophagy may be associated with an adaptive response that suppresses cell death and subsequently improves renal tubular cell survival during kidney injury^50^. Various signaling pathways are known to influence autophagy-linked molecular pathways. CP-induced pathological outcomes have been negatively associated with protective autophagy activation via metabolic reprogramming-linked biochemical signaling mediators, including 5′-adenosine monophosphate-activated protein kinase alpha and sirtuin-3, during acute renal injuries^51,52^. Moreover, the autophagic machinery sequesters damaged lysosomes in kidney cells, which are engulfed via autophagosome formation, resulting in lysosomal homeostasis and healthy cellular integrity during AKI^53^. In addition, the autophagy-linked intracellular degradation process plays crucial roles in mucin-secreting intestinal cells to cope with the unfolded protein stress responses^54,55^. Excess levels of mucin biosynthesis with defective autophagy pathway may disrupt the intestinal homeostasis. In contrast to protective autophagy, autophagy signaling has been associated with detrimental effects via proinflammatory responses in an endotoxin-associated kidney injury model^56^. Notably, autophagy can afford protection against a mild degree of renal ischemia-reperfusion injury; however, this effect is not observed in cases of severe injury^57^. Furthermore, persistent activation of autophagy in the kidney proximal tubules was shown to stimulate renal interstitial fibrosis in a murine model^58^. Therefore, the precise contribution of renal and intestinal cells during autophagy needs to be thoroughly assessed depending on disease severity and progression.

In the present study, autophagy signaling was associated with PKR-mediated ISR in the acute renal injury model using CP treatment. CP induces genotoxicity by forming DNA adducts, which interferes with genetic replication and cellular stress responses. However, CP-induced pathological outcomes can be associated with multiple mechanisms, including the blockade of RNA synthesis or its direct binding to RNA^35–37^. CP-DNA adducts were found to exhibit high binding activity to high mobility group domain proteins such as the human upstream binding factor, which can interfere with binding to the ribosomal RNA (rRNA) gene promoter^35,37^. Moreover, CP directly intercalates ribosomes and mRNA, leading to ribosomal stalling, as well as suppressed global translation^36^. Stress-driven ribosomal stalling triggers eIF2α-mediated global translational inhibition via PKR, a primary biochemical pathway in ISR^27,59–61^. PKR-linked ISR can be activated by diverse internal and external stressors, including viral infection, specific translational inhibition, oxidative and ER stress, growth factor deprivation, and bacterial infection^27,62^. Notably, ribosomal stress-linked PKR activation is a potent mechanism involved in inflammatory and chronic disorders via imbalanced cytokine and growth factor profiles^63–65^. Moreover, PKR-linked events can be associated with generalized renal pathological stress rather than chemical-specific renal toxicity, as patients with AKI and CKD displayed such links regardless of platinum exposure (Fig. 1). Therefore, extensive molecular evidence is needed to establish whether stress-responsive signaling facilitates adverse outcomes or restores biological homeostasis in diverse kidney injuries.

In conclusion, stress-responsive Gdf15 was proven to be an adaptation molecule against acute renointestinal injuries. Gdf15 expression was dependent on signaling activation mediated by PKR-linked ISR, which was verified by clinical transcriptome analysis in patients with renal injuries. In particular, stress-responsive Gdf15 was involved in the maintenance of gut mucosal and renal cell integrity. Mechanistically, Gdf15 controlled acute renointestinal distress by facilitating mucin production and cellular protection via the reorganization of cell-protective autophagic signaling. Moreover, Gdf15 was associated with the regulation of the mucus-degrading bacterial community. Collectively, stress-responsive endogenous adaptation responses could counteract pathological processes in the ISR-Gdf15-autophagy network signaling axis, thereby paving a new strategy for the development of predictive biomarkers and interventions against renointestinal distress.

## Methods

### Analysis using clinical transcriptome-based datasets

Renal gene expression was assessed in patients with AKI (gse30718, *n* = 47) or CKD (gse66494, *n* = 61). Gene expression analysis of human kidneys with AKI is limited since such kidneys are seldom biopsied^66^. Given the limited supply of kidney biopsy samples from patients with AKI, patients with kidney explants were followed up as models of human AKI^66^. Since all subjects with kidney transplants experience AKI, early kidney transplants without rejection are an excellent model for human AKI. Biopsies from transplants with AKI (gse30718) were compared with those from the pristine protocol biopsies of stable transplants.

Patients with CKD display irreversible and progressive loss of nephrons, followed by glomerular fibrosis, tubular atrophy, and tubulointerstitial fibrosis, which are commonly observed features of human progressive renal diseases, irrespective of the initial etiologies^67^. Biopsy specimens from 48 patients with histopathologically confirmed CKD (gse66494) were analyzed to identify genes responsible for tubulointerstitial fibrosis and tubular cell injury using two independent discovery and validation processes^67^. The patients exhibited various histological types of renal diseases including IgA nephropathy (*n* = 15), membranous nephropathy (*n* = 7), lupus nephritis (*n* = 6), minimal change nephrotic syndrome (*n* = 3), membranoproliferative glomerulonephritis (*n* = 3), amyloidosis (*n* = 3), antineutrophil cytoplasmic antibody-related glomerulonephropathy (*n* = 2), diabetic nephropathy (*n* = 2), and other nephropathies (*n* = 6).

### Cell culture

In the present study, we employed the human intestinal epithelial cell line HCT-8 and human kidney cells (HK-2 and HEK293). The mycoplasma-free cell lines were purchased from American Type Culture Collection and maintained in RPMI 1640 or DMEM medium (Welgene) supplemented with 10% (v/v) heat-inactivated fetal bovine serum (Welgene), 50 U/mL penicillin, and 50 µg/mL streptomycin (Welgene) at 37 °C in a humidified 5% CO_2_ incubator. Cells were seeded at a density of 5×10^5^ cells in a 60 mm dish, and the culture medium was replenished when cells achieved confluency. After replenishing the medium for 12 h, CP (10 μmol/L) in normal saline was added to the cell culture for 24 or 48 h. Subsequently, cells were harvested for protein and RNA extraction. CP powder was purchased from Sigma-Aldrich (#PHR1624), and stock solutions were prepared in normal saline. Cell counting and viability were assayed using the dye exclusion test with Trypan blue dye (Merck) with a hemocytometer.

### Chemically induced acute renointestinal injury models

The procedure was based on the previously published method^68^. In detail, C57BL/6 mice (6-week-old male, average weight 16–18 g) were purchased from Jackson Laboratories (Bar Harbor, ME, USA), and Gdf15 KO C57BL/6 mice were kindly provided by Dr. Se-Jin Lee (Johns Hopkins University, Baltimore, MD, USA). All mice of the same age were placed in one cage and randomly transferred to different cages without bias. Mice were randomly assigned to specific treatment groups. The mice were acclimatized for 14 days before experimentation and maintained at 22 ± 2 °C under 45–55% relative humidity, with a 12/12 h light/dark cycle. Three mice were housed per cage and provided sufficient food and water in environmentally protected cages, comprising a transparent polypropylene body and stainless-steel top cover. Chemotherapy-induced AKI was induced by an intraperitoneal injection of cisplatin (20 mg/kg). Eight-week-old wild-type and Gdf15 KO mice were treated with vehicle or CP (20 mg/kg, intraperitoneally) for 72 h (*n* = 4 − 5). To induce DSS-induced colitis and renal complications, seven-week-old male mice were treated with 3% DSS (molecular weight 36,000–50,000 Da; MP Biomedical, Solon, OH, USA) *ad libitum* in drinking water for eight days. Two days later, the mice were sacrificed under deep ether anesthesia.

### Histopathology

The procedure was based on the previously published method^68,69^. Briefly, kidney sections were stained with PAS, and microphotographs of prepared sections were obtained using an inverted microscope (Nikon-Eclipse Ts2R, Tokyo, Japan). The images obtained were quantified using Adobe Photoshop CS6 and Multi Gauge V3.0. One portion of the harvested kidney was fixed in 4% PFA at 4 °C, embedded in paraffin wax, and stained with PAS; the other part was segmented into two portions for RNA and protein isolation, snap-frozen in liquid nitrogen at -120 °C for 10–15 min, and homogenized on the day of organ harvest. The degree of renal tubular injury was expressed as the proportion of damaged renal tubules^70^. For statistical analysis, the visual fields of each specimen were randomly selected, and tubule diameters and lumen diameters were photographed under the microscope, and the lesions were statistically analyzed by ImageJ software. Tubule diameters were measured from the basal membrane across the narrowest segment of a tubule to the basal membrane of the opposite side. Lumen diameters were determined by measuring the distance between the apical membranes across the narrowest portion of a tubule. The levels of tubular vacuolization were assessed by grading levels of vacuoles affecting proximal tubules and extending to the basement membrane^71^. The cystic index was calculated as the percentage of cystic area relative to the total kidney area^72^.

The procedure was based on the previously published method^68,69^. For gut analysis, the small intestine was immediately removed, fixed in Carnoy’s solution, and embedded in paraffin. The sections were dewaxed using xylene, rehydrated using a series of graded alcohol solutions, and stained with hematoxylin and eosin (H&E) using an established laboratory protocol to reveal histopathological lesions. Villous and crypt lengths of the jejunum and ileum were measured using a micrometer. At least nine villi from each section were measured and averaged for each group. Morphological evaluation was performed in a blinded manner using well-established criteria. In brief, H&E-stained cross‐sections of small intestinal tissues were scored on a 0–4 scale to determine histopathological severity based on the following criteria: 0, no change from normal tissue; Grade 1, mild inflammation present in the mucosa, comprising mainly mononuclear cells, with little epithelial damage; Grade 2, multifocal inflammation exceeding a Grade 1 score, with mononuclear and few polymorphonuclear cells (neutrophils), crypt glands pulled away from the basement membrane, mucin depletion from goblet cells, and the epithelium occasionally pulled away from the mucosa into the lumen; Grade 3, multifocal inflammation exceeding a Grade 2 score, including mononuclear cells and neutrophils progressing into the submucosa, crypt abscesses present with increased mucin depletion, and presence of epithelial disruption; Grade 4, absence of crypts, severe mucosal inflammation mainly composed of neutrophils, and epithelium no longer present or completely detached. For each mouse, the average of three fields of view was examined per small intestine sample. All evaluators were blinded to the present experimental information.

### Alcian blue staining

The procedure was based on the previously published method^68,69^. The prepared tissue sections were deparaffinized in xylene for 10 min, rehydrated in ethanol (100, 90, 80, 70, and 50%) for 3 min each, and then placed in distilled water for 10 min. AB 8GX (1% [w/v] alcian blue 8GX, Biosesang, Seoul, Korea) solution was applied to sections for 30 min at 25 °C, followed by a 2 min wash under running tap water. Counterstaining was performed for 5 min with 0.1% (w/v) nuclear Fast Red, followed by washing for 2 min under running tap water. Subsequently, the stained sections were dehydrated in two changes of 95% ethanol and two changes of absolute alcohol. The dried sections were cleared using xylene for a few minutes and then mounted using a synthetic mountant (Thermo Fisher Scientific, Seoul, Korea).

### Tissue detection of the mucosal microbiota

Gram staining was performed as follows^45^: tissue sections were deparaffinized in xylene, rehydrated in ethanol, incubated in dH_2_O for 5 min, stained for 1 min using crystal violet, and then washed under tap water. Slides were then stained with Gram’s iodine for 1 min, rinsed under tap water, dipped in 95% ethyl alcohol for 5–10 s, and rinsed again under tap water. The slides were stained again for 1–2 min in safranin and rinsed under tap water. Stained sections were rapidly dehydrated in two changes of 95% ethanol, followed by two changes of absolute alcohol, cleared in xylene, and mounted in a synthetic mountant (Thermo Fisher Scientific, Korea). The safranin stains decolorized gram-negative cells red/pink, while the gram-positive bacteria remained blue.

In situ detection using probes for 16 S rRNA was performed as follows: tissues were deparaffinized with xylene and rehydrated through an ethanol gradient to water. Sections were incubated with a universal Cy3-labelled bacterial probe directed against the 16 S rRNA gene (5′-GCT GCC TCC CGT AGG AGT-3′) at 50 °C overnight and counterstained with DAPI for nuclear staining.

### Serum creatinine and urea nitrogen levels

The procedure was based on the previously published method^68^. The creatinine level was measured using the Creatinine Serum Detection Kit (KB02-H2, Arbor Assays, MI, USA) according to the manufacturer’s instructions. Briefly, mice were anesthetized with 30 µL of isoflurane (Hana Pharm Co., Seoul, Korea), and blood was collected by performing a retro-orbital sinus puncture into a 1.5 mL tube containing 10 μL of 0.5 M EDTA, which was subsequently centrifuged at 1000× *g* for 15 min to separate the plasma, followed by storage at -80 °C. Before performing the assay, all samples were centrifuged at 18,341× *g* for 15 min. Then, standard solutions (25 μL) with known concentrations of creatinine stock, blood samples, or water (blank) diluted with 25 μL of assay diluent were mixed with 100 μL of DetectX Creatinine Reagent in a 96-well microplate and incubated for 30 min. Optical density was measured using a microplate reader at a wavelength of 490 nm. BUN was measured using a urea nitrogen colorimetric detection kit (KB024-H1, Arbor Assays) according to the manufacturer’s instructions. Briefly, obtained plasma was centrifuged at 18,341× *g* for 10 min and diluted in distilled water (1:20). Then, 50 µL of samples or appropriate standards were pipetted into a 96-well plate in duplicates. Next, 75 µL of color reagents A and B was added to each well using a repeater pipette. The plate was incubated at 25 °C for 30 min, and optical density was measured at 450 nm.

### Western blot analysis

Briefly, kidney samples were lysed in 1 mL of modified lysis buffer (1% [w/v] sodium dodecyl sulfate [SDS], 1.0 mM sodium orthovanadate, and 10 mM Tris; pH 7.4) containing stainless-steel beads (5 mm) and homogenized for 3 min using a TissueLyser II (Qiagen). The lysates were clarified by centrifugation at 13,475*× g* for 10 min at 4 °C and quantified using a BCA Protein Assay Kit (Pierce, Rockford, IL, USA). Equal amounts of protein (30 μg) were separated using SDS-polyacrylamide gel electrophoresis (8% gel for anti-PARP 1/2, 15% gel for anti-cleaved caspase-3) in a Bio-Rad gel mini electrophoresis system (Bio-Rad, Hercules, CA, USA). The proteins were transferred onto a polyvinylidene difluoride membrane (0.45 μm; EMD Millipore Corporation, Billerica, MA, USA) and then blocked with 5% skim milk in Tris-buffered saline plus 0.1% Tween (TBST) for 1 h. Subsequently, the membranes were incubated with the desired primary antibody overnight at 4 °C. After washing three times with TBST, the blots were incubated with horseradish peroxidase (HRP)-conjugated secondary antibody for 2 h, followed by washing with TBST three times for 30 min. Antibody binding was detected using an enhanced chemiluminescence substrate (ELPIS Biotech, Daejeon, Korea). The following antibodies were used for western blotting: mouse monoclonal anti-β-actin (1:1000; SC4778, Santa Cruz Biotechnology, Santa Cruz, CA), rabbit polyclonal anti-PARP1/2 (1:1000; SC7150, Santa Cruz Biotechnology), and rabbit polyclonal anti-Gdf15 (Abclonal, Woburn, MA, USA). The secondary antibodies used were HRP-conjugated goat anti-rabbit IgG, polyclonal antibody (ADI-SAB-300-J, Enzo Biochem Inc. Farmingdale, NY, USA), and goat anti-mouse IgG (BML-SA204, Enzo Biochem Inc. Farmingdale, NY, USA).

### Conventional and reverse transcription-quantitative polymerase chain reaction

The procedure was based on the previously published method^68,69^. Frozen tissues were homogenized in 1 mL RiboEx solution (GeneAll Biotechnology, Seoul, Korea) containing stainless-steel beads (5 mm) for 3–6 min using a TissueLyser II (Qiagen). Total RNA was extracted using RiboEx (GeneAll Biotech, Seoul, South Korea), according to the manufacturer’s instructions. Subsequently, RNA (500 ng) from each sample was transcribed to cDNA using a TOPscript RT DryMIX cDNA synthesis kit (Enzynomics, Daejeon, Korea). cDNA was amplified using N-Taq DNA polymerase (Enzynomics) in a MyCycler Thermal Cycler (Bio-Rad) using the following parameters: denaturation at 95 °C for 5 min, followed by 25 cycles of denaturation at 95 °C for 10 s, annealing at 60 °C for 15 s, and elongation at 72 °C for 30 s. The following primers were used for PCR: human *GAPDH*, 5′-TCA ACG GAT TTG GTC GTA TT-3′ and 5′-CTG TGG TCA TGA GTC CTT CC-3′; human *MUCIN2*, 5′-TTA CCC ACT GCG TGG AAG AC-3′ and 5′-GCA TTC CCG TGA ACA CAC AC-3′; human *MUCIN4*, 5′-GAC GAC TTC AGG ATG CCC AA-3′ and 5′-AGG GCC AGG GTG TCA TAG AT-3′; human *Gdf15*, 5′-ACG CTA CGA GGA CCT GCT AA-3′ and 5′-AGA TTC TGC CAG CAG TTG GT-3′; human *GABARAPL1*, 5′- AGG AGG ACC ATC CCT TTG AGT A-3′ and 5′- TCC TCA GGT CTC AGG TGG ATT-3′; human *PPARγ*, 5′- TTC AGA AAT GCC TTG CAG TG-3′ and 5′- CAC CTC TTT GCT CTG CTC CT-3′. An aliquot of each polymerase chain reaction (PCR) product was subjected to 1.2% (w/v) agarose gel electrophoresis and was visualized by staining with ethidium bromide. For real-time PCR, cDNA was amplified using SYBR green (SG, TOPreal™ qPCR 2X PreMIX, Enzynomics), performed with Rotor-Gene Q (Qiagen, Hilden, Germany) using the following parameters: denaturation at 94 °C for 2 min, followed by 40 cycles of denaturation at 98 °C for 10 s, annealing at 59 °C for 30 s, and elongation at 98 °C for 45 s. Each sample was evaluated in triplicates to ensure statistical robustness. Relative quantification of gene expression was performed using the comparative Ct method, in which the Ct value was defined as the point at which a statistically significant increase in fluorescence was observed. The number of PCR cycles (Ct) required for the fluorescence intensities to exceed a threshold value immediately above the background level was calculated for the test and reference reactions. GAPDH was used as an internal control for all experiments.

### Confocal microscopy

Cells transfected with pDEST-CMV mCherry-GFP-LC3B WT (a gift from Robin Ketteler, Addgene plasmid # 123230, Watertown, MA, USA) were exposed to different treatments. Cells were washed with PBS and then the coverslips were carefully placed and slight pressure was applied to remove the remaining bubbles. Confocal images were obtained with an Andor BC43 Benchtop confocal microscope (Andor, Belfast, UK) at the single-line excitation (529 nm for GFP, or 600 nm for mCherry). Images were acquired with the Fusion software (Andor) and processed with the Imaris software (Andor).

### Analysis of the autophagy regulatory network (ARN)

PPI network analysis was performed using the search tools for the retrieval of interacting genes (STRING) (https://string-db.org/) and ARN databases, which cover various evidence-based autophagy machinery-linked components, their regulators, transcription factors, miRNA regulators, and signaling network connectors in multi-layered illustration^40^. The betweenness centrality of node *x* was calculated using function g(*x*).

1 $${{{{{\rm{g}}}}}}(x)={\sum }_{s\ne x\ne t}{{{{{{\rm{\sigma }}}}}}}_{{st}}(x)/{{{{{{\rm{\sigma }}}}}}}_{{st}}$$

where ${{{{{{\rm{\sigma }}}}}}}_{{st}}$ is the total number of shortest paths from node *s* to node *t*, and ${{{{{{\rm{\sigma }}}}}}}_{{st}}\left(x\right)$ is the number of paths that pass through node *x* (not where *x* is an endpoint).

### Stool sample collection and DNA extraction

The procedure was based on the previously published method^68,69^. Eight-week-old wild-type and Gdf15 KO mice were treated with vehicle or CP (20 mg/kg, intraperitoneally) for 72 h (*n* = 4 − 5). Fecal samples (5 − 6 pieces; 100 mg) were collected before animal sacrifice. The collected stool samples were stored at -150 °C before DNA extraction. Microbial DNA was extracted and purified from 100 mg of the fecal sample using the Exgene Stool DNA mini kit (GeneALL, Seoul, Korea), according to the manufacturer’s instructions. The extracted DNA was quantified using a Qubit fluorometer and high-sensitivity dsDNA reagent kit (Invitrogen, Carlsbad, CA, USA).

### Amplification of 16 S rRNA genes and library preparation

The procedure was based on the previously published method^68,69^. Thirteen DNA samples were pooled as treatment groups for 16 S metagenomic library preparation. The V3–V4 region of the 16 S rRNA gene was PCR-amplified with a primer set (341 F, 5′-CCTACGGGNGGCWGCAG-3′; 805 R, 5′-GACTACHVGGGTATCTAATCC-3′) and Illumina sequencing adaptors (Illumina Inc.) using the KAPA HiFi HotStart Ready Mix (KAPA Biosystems, Wilmington, WA, USA) under the following cycling conditions: initial denaturation 95 °C for 3 min, followed by 25 cycles of denaturation at 95 °C for 30 s, annealing at 55 °C for 30 s, extension at 72 °C for 30 s, and a final extension at 72 °C for 5 min. After amplicon purification using AMPure® XP beads (Agencourt Biosciences, Beverly, MA, USA), the PCR products were verified for library size using a BioAnalyzer (Agilent Technologies, Palo Alto, CA, USA), and the quantity was measured using a Qubit fluorometer. The PCR amplicon was then subjected to indexing PCR using the Nextera XT Index Kit (Illumina, Inc.). The PCR cycling conditions were as follows: 95 °C for 3 min, followed by eight cycles of denaturation at 95 °C for 30 s, annealing at 55 °C for 30 s, extension at 72 °C for 30 s, and a final extension at 72 °C for 5 min. The indexed PCR amplicons were purified using AMPure® XP beads, verified for size using a bioanalyzer, and quantified using a Qubit fluorometer. The quantified amplicons were diluted to 4 nM and pooled for sequencing on an Illumina iSeq platform (Illumina, Inc.), targeting 2× 150 bp paired-end sequence reads.

### Microbiome data processing

The procedure was based on the previously published method^68,69^. All sequences were quality-filtered, and primers were trimmed using Trimmomatic (v0.39)^73^ with the following parameters: LEADING:3 TRAILING:3 MINLEN:36 SLIDINGWINDOW:4:15. The read pairs that passed the quality filter were further analyzed using the Quantitative Insights into Microbial Ecology 2 (QIIME2, v2020.2) pipeline^74^. Thirty-four bases of the reverse reads were truncated for quality improvement using the DADA2 algorithm^75^. The read pairs were then joined, denoised, and dereplicated, and chimeras were removed. Detailed data were used to refer to ASVs. Representative 16 S rRNA sequences were assigned to taxonomic groups using the QIIME2 naive Bayes classifier, trained on 99% operational taxonomic units (OTUs) and the primer region from the SILVA rRNA database (v138)^76^. Taxonomic classification was visualized using the QIIME2 taxon barplot plugin. Representative 16 S rRNA sequences were subjected to masked multisequence alignment using MAFFT^77,78^. A phylogenetic tree was constructed using FastTree^79^. A heatmap depicting the percentage abundance of OTUs, the relative abundance of which was in the top 30 in any sample, was generated using the R package QIIME2R (v0.99.22). A phylogenetic tree based on the abundant OTUs was constructed and visualized using the neighbor-joining algorithm with Jukes-Cantor correction using MEGA X^80^. The 16 S rRNA sequence dataset was further analyzed using PICRUSt2 to predict metagenome-associated functions, such as enzymes and pathways related to mucin metabolism.

### Statistics and Reproducibility

Statistical analysis was performed using the GraphPad Prism 6 software (GraphPad Software, La Jolla, CA, USA). The *Student*’s t-test was used for the comparative analysis of two data groups. To compare multiple groups, data were subjected to analysis of variance (ANOVA) with the *Newman–Keuls* method performed as a posthoc ANOVA assessment. Pearson’s correlation analysis was performed to determine the correlation coefficient (R) of clinical datasets. All evaluations are representative of two or three independent experiments. Details of the number of biological replicates and assays are provided in figure legends.

### Study approval

This animal study was approved by the Pusan National University Institutional Animal Care and Use Committee (PNU-IACUC, Busan, Korea) (PNU-2019-2365) and was performed under the Declaration of Helsinki and with the Guide for the Care and Use of Laboratory Animals as adopted and promulgated by the United States National Institutes of Health.

### Reporting summary

Further information on research design is available in the Nature Portfolio Reporting Summary linked to this article.

## Supplementary information

Moon_Peer Review File

Supplementary Information

Description of Additional Supplementary Files

Supplementary Data

Reporting Summary

## Data Availability

Source data are provided in Supplementary Data. The data are available upon request from the authors. The data supporting the findings of this study are available from the corresponding author upon reasonable request. Some data may not be available owing to privacy or ethical restrictions. The uncropped Western blots can be found in the supplementary figure s5.
