# Supplementary material for: Stress-responsive Gdf15 counteracts renointestinal toxicity via autophagic and microbiota reprogramming
Source: Commun Biol. 2023 Jun 3;6:602. doi: 10.1038/s42003-023-04965-1 (PMC10239500; doi:10.1038/s42003-023-04965-1)
Supplement: Supplementary file 2 — Supplementary Information [file 42003_2023_4965_MOESM2_ESM.pdf]

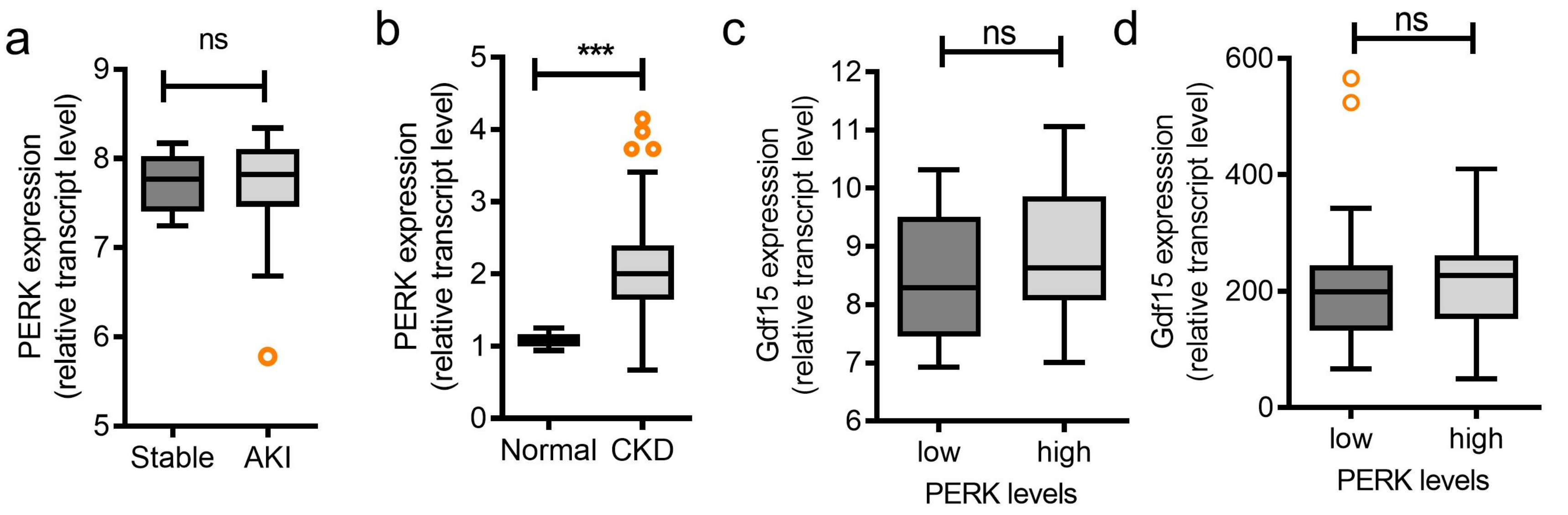

Supplementary figure s1. Expression of EIF2AK3 (PERK) and Gdf15 in patients with acute kidney inj ury (GSE30718, a, c) or chronic kidney disease (GSE66494, b, d). c-d. Based on PERK levels, we select ed the 20 highest and 20 lowest samples, followed by a comparison of Gdf15. The results are shown as a plot with Tukey whiskers and outliers (orange circles). The asterisks (∗) indicate significant differences b etween the two groups (***$p < 0.001$).

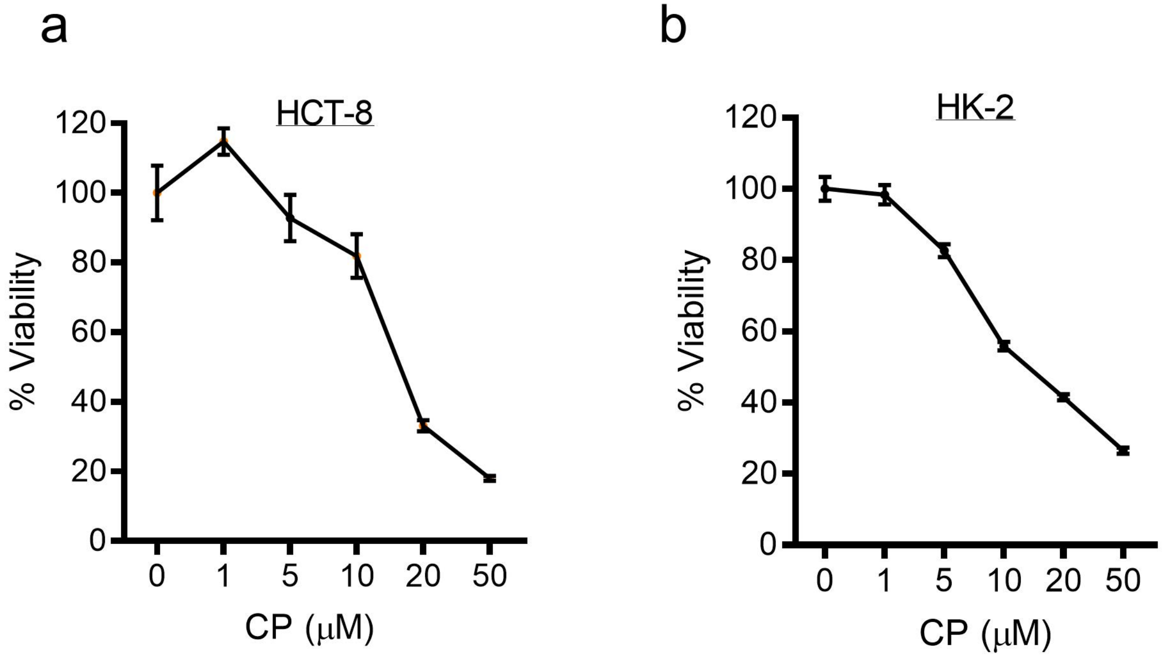

Supplementary figure s2. HCT-8 (a) and HK-2 cells (b) were treated with different doses o f CP for 24 h before MTT viability assay.

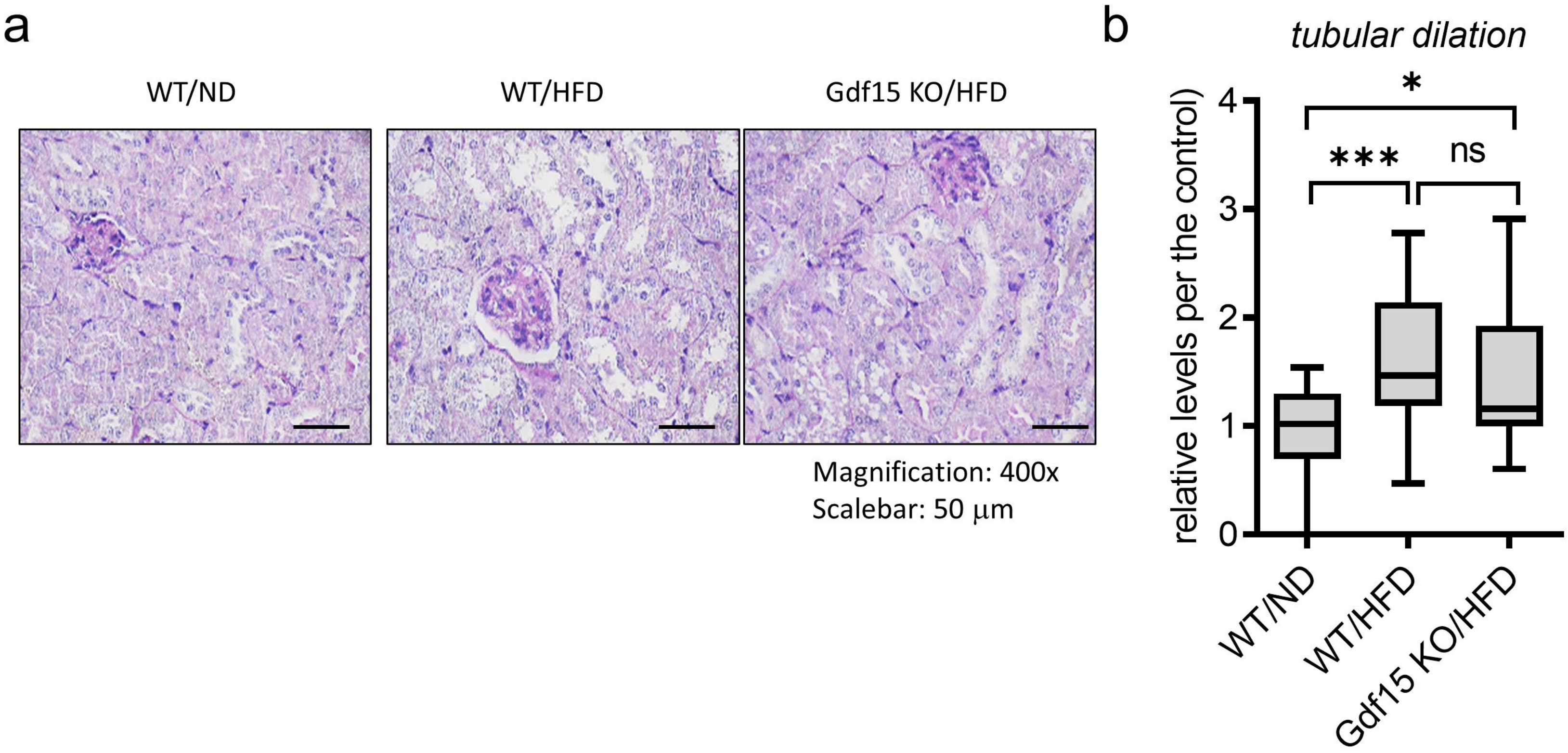

Supplementary figure s3. Three-week-old wild-type and Gdf15 knockout (KO) male mice were fed with the normal diet (ND) and high fat diet (HFD) for 14 weeks (n=4−6). Histological examination of PAS-stained kid ney sections (a, Magnification, 400×; Scale bars(s), 50 μm). Quantitative analysis of tubular dilation (b), sho wn as a plot with Tukey whiskers, and the asterisks (∗) indicating significant differences between groups (∗p < 0.05, ∗∗∗p < 0.001 using a two-tailed unpaired Student's t-test). Gdf15, growth differentiation factor 15.

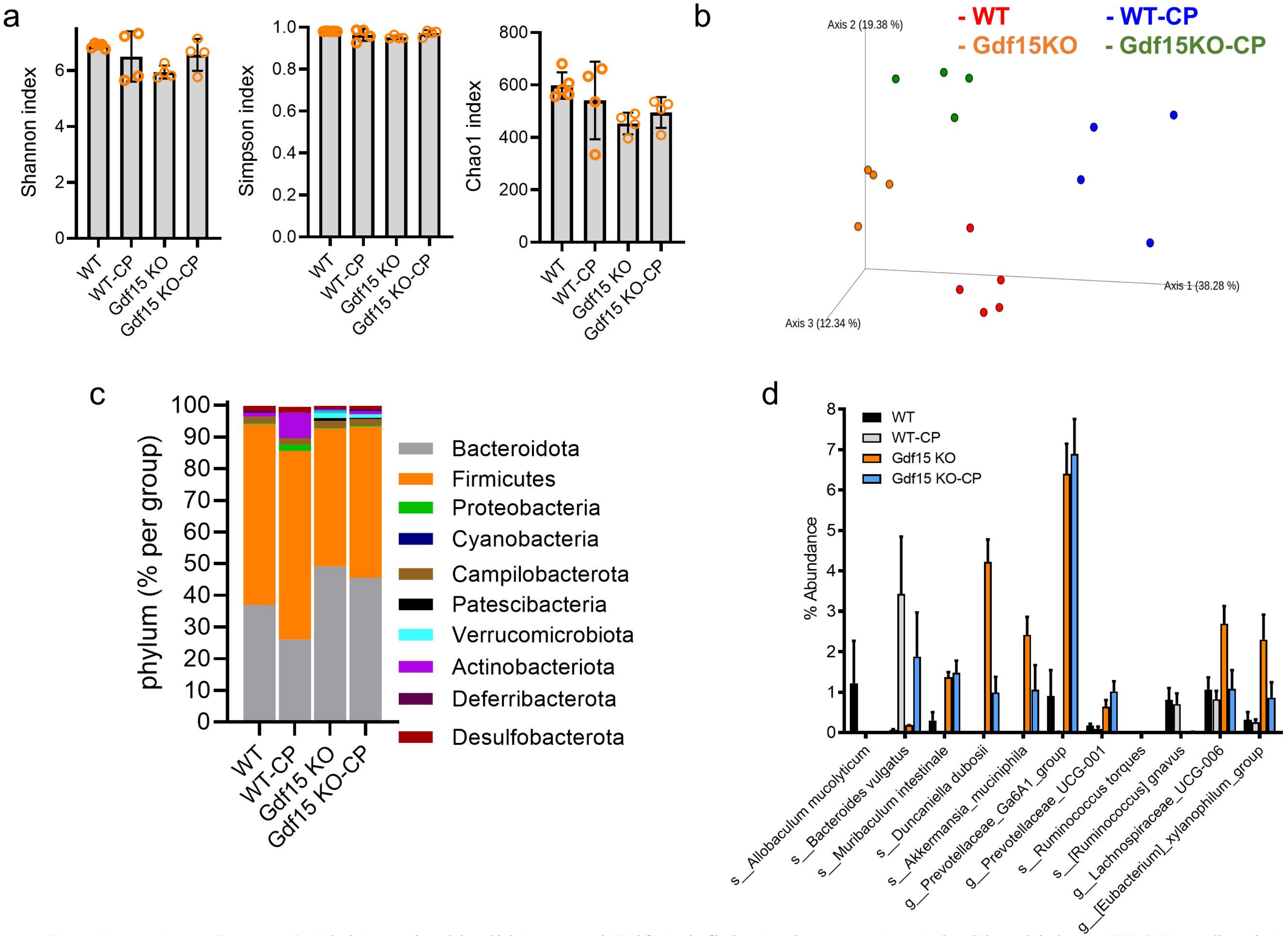

Supplementary figure s4. Eight-week-old wild-type and Gdf15-deficient mice were treated with vehicle or CP (20 mg/kg, int raperitoneal) for 72 h (n = 4−5). The fecal bacteria were subjected to 16S rRNA analysis for the determination of the phylogen etic composition. **a**. Shannon, Simpson, and Chao1 indexes for the alpha diversity. **b**. Beta diversity from 16S rRNA analysis. **c**. phylogenetic composition at the phylum level. **d.** Abundance of potent mucin foragers in each treatment group.

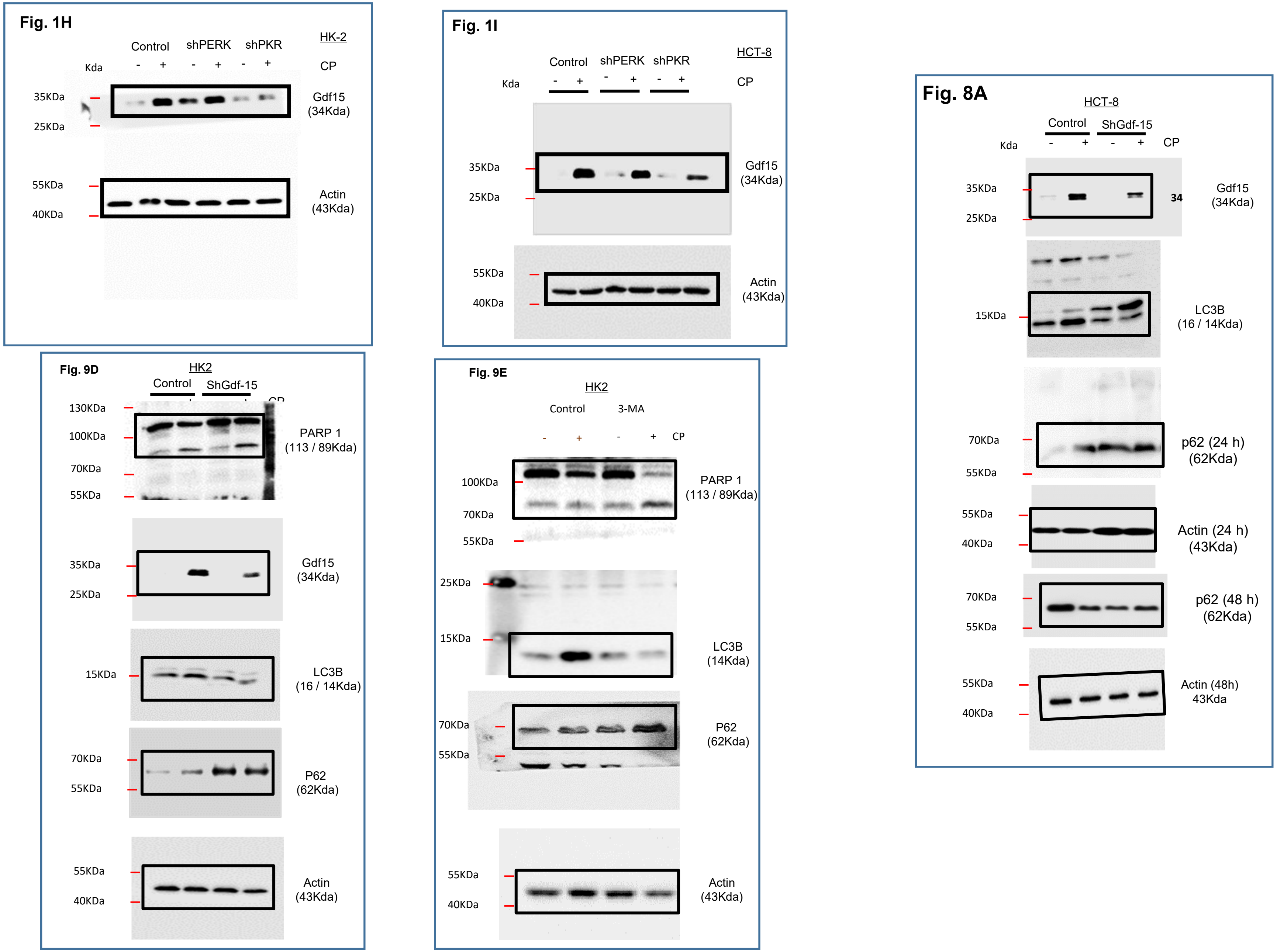

Supplementary figure s5. The uncropped Western blots for all data in the main figure. Since size marker proteins can not be visible under the chemiluminescence detector, the size of each detected protein was confirmed by matching with the size markers on the PVDF membrane.
