# Supplementary material for: Stress-responsive Gdf15 counteracts renointestinal toxicity via autophagic and microbiota reprogramming
Source: Commun Biol. 2023 Jun 3;6:602. doi: 10.1038/s42003-023-04965-1 (PMC10239500; doi:10.1038/s42003-023-04965-1)
Supplement: Supplementary file 1 — Moon_Peer Review File [file 42003_2023_4965_MOESM1_ESM.pdf]

nature portfolio

Peer Review File

Stress-responsive Gdf15 Counteracts Renointestinal Toxicity via Autophagic and Microbiota Reprogramming

Open Access This file is licensed under a Creative Commons Attribution 4.0 International License, which permits use, sharing, adaptation, distribution and reproduction in any medium or format, as long as you give appropriate credit to the original author(s) and the source, provide a link to the Creative Commons license, and indicate if changes were made. In the cases where the authors are anonymous, such as is the case for the reports of anonymous peer reviewers, author attribution should be to 'Anonymous Referee' followed by a clear attribution to the source work. The images or other third party material in this file are included in the article's Creative Commons license, unless indicated otherwise in a credit line to the material. If material is not included in the article's Creative Commons license and your intended use is not permitted by statutory regulation or exceeds the permitted use, you will need to obtain permission directly from the copyright holder. To view a copy of this license, visit http://creativecommons.org/licenses/by/4.0/.

Reviewers' comments:

Reviewer #1 (Remarks to the Author):

Journal : Communications Biology Peer Review
Authors : Navin Ray, Hoyung Jung, and Yuseok Moon
Title : Stress-responsive Growth Differentiation Factor 15 Counteracts Acute Renointestinal Distress via Mucoprotective Reprogramming.
Communications Biology. 2022
Manuscript ID : COMMSBIO-22-3243

In this report, Ray and colleagues examined the role of Growth differentiation factor 15 (Gdf15) in stress response mechanisms of renal injuries and intestinal mucosal injuries. injuries. They performed trnscriptome analysis of patients with acute kidney injury (AKI) or chronic kidney disease (CKD) and identified protein kinase R (PKR) positively associated with Gdf15 expression in patients with renal injury.
They used two animal models, cisplatin-indued AKI mice and DSS-induced colitis model mice with wild type mice and Gdf15-deficent mice and demonstrated protective effects of Gdf15 in renointestinal distress.
They also used HK2 cells and HCT-8 cells for culture model of Kidney and gut, respectively. Cisplatin treatment increased Gdf15 expression in both HK2 cells and HCT-8 cells in stress-related mammalian eIF2ɑ kinase, i.e. protein kinase R(PKR) dependent manner. They reported one of the protective mechanism of Gdf15 was associated autophagy.

It was interesting that Gdf15 might have therapeutic potential to renal and gut mucosal injuries via stress-related mammalian eIF2ɑ kinase and autophagy pathway.
But several additional experiments should be performed to improve the quality of this paper for for publication.

Major
1) Authors performed trnscriptome analysis of both AKI and CKD patients and got the similar stress induced transcriptional changes and started the analysis of Gdf15 role inanimal and cell culture mode. But only cisplatin-induced mice AKI model was performed in kindey injury. The role of Gdf15 in CKD models, such as 5/6-nephrectomixed-mice, adenin-induced CKD mice. Also the unilateral ureteral obstruction(UUO)-mice model of Gdf15-deficient mice might be useful to investigate the Gdf15 role in renal fiborosis.

2) Authors investigated the autophagy pathway in the renoprotective and mucosa-protective mechanisms of Gdf15. But only the LC3B protein expression was reported as the marker of autophagy pathway. It should be better to analyze the p62 expression and visualized autophagosome dynamics in cell culture model to confirm the autophagy dynamics in cisplatin-induced HK2/HCT-8 cell culture models

3) The data of autophagy pathway cisplatin-indued AKI mice and DSS-induced colitis model mice are desirable to confirm the role Gdf15- autophagy pathway autophagy pathway.

Reviewer #2 (Remarks to the Author):

It was my pleasure to read the manuscript "Stress-responsive Growth Differentiation Factor 15 Counteracts Acute Renointestinal Distress via Mucoprotective Reprogramming" by Moon et al.

Overall the paper is well written. The topic is important and interesting to the nephrologic community. The paper provides new mechnistical insights into the (patho-)physiological role of gdf15 during renal/intestinal injury.

I have only minor remarks:
Because reasons for CKD and AKI and patient cohorts can largely differ, it would be interesting to the reader to have a little bit more information on the analyzed cohorts (GSE30718 and GSE66494).

Sometimes I read GDF15 sometimes gdf15 (e.g. Fig. 1, 3). Please unify.

Fig. 2 legend is mixed up 2B -> 2C etc. Moreover, not all parts of Fig. 2 are covered by the legend.

The criteria used (grading) for the histological grading (2F-H) are not clear to me.

Fig 7A is the number under HCT-8 control (+) row 12.28 or 2.28?

MS ID#: **COMMSBIO-22-3243**

MS TITLE: "**Stress-responsive Growth Differentiation Factor 15 Counteracts Acute Renointestinal Toxicity via Autophagic and Microbiota Reprogramming**"

Dear Referees,

We really appreciate your kind comments on my manuscript. It was very nice chance to look deeply again into the results and scientific and translational meaning with your precious comments and we did best to come up with every requirement from the reviewers. We will follow up each comment and suggest responses point-by-point to improve experimental results and resubmit the revised manuscript. All edited parts were written in the red color.

Sincerely yours,

Yuseok Moon, corresponding author

<u>Responses to Reviewer #1' comments</u>

In this report, Ray and colleagues examined the role of Growth differentiation factor 15 (Gdf15) in stress response mechanisms of renal injuries and intestinal mucosal injuries. injuries. They performed trnscriptome analysis of patients with acute kidney injury (AKI) or chronic kidney disease (CKD) and identified protein kinase R (PKR) positively associated with Gdf15 expression in patients with renal injury.

They used two animal models, cisplatin-indued AKI mice and DSS-induced colitis model mice with wild type mice and Gdf15-deficent mice and demonstrated protective effects of Gdf15 in renointestinal distress. They also used HK2 cells and HCT-8 cells for culture model of Kidney and gut, respectively. Cisplatin treatment increased Gdf15 expression in both HK2

cells and HCT-8 cells in stress-related mammalian eIF2α kinase, i.e. protein kinase R(PKR) dependent manner. They reported one of the protective mechanism of Gdf15 was associated autophagy.

It was interesting that Gdf15 might have therapeutic potential to renal and gut mucosal injuries via stress-related mammalian eIF2α kinase and autophagy pathway. But several additional experiments should be performed to improve the quality of this paper for for publication.

1) Authors performed trnscriptome analysis of both AKI and CKD patients and got the similar stress induced transcriptional changes and started the analysis of Gdf15 role in animal and cell culture mode. But only cisplatin-induced mice AKI model was performed in kindey injury. The role of Gdf15 in CKD models, such as 5/6-nephrectomixed-mice, adenin-induced CKD mice. Also the unilateral ureteral obstruction (UUO)-mice model of Gdf15-deficient mice might be useful to investigate the Gdf15 role in renal fiborosis.

Response: As a reviewer pointed out, we additionally performed renal injury in non-cisplatin model (**Figure 4C-4E**). In addition to the main impacts on the gut ulcerative injury, DSS treatment can trigger mild renal injury, which was aggravated in the Gdf15 knockout mice, including glomerular injuries, including morphological shrinkage and neutrophil infiltration. We used two animal models of gut-kidney axis using cisplatin and DSS treatment, but we additionally tried a representative model of chronic kidney disease using high fat diet (**supplementary figure S3**). In this model, we could not find notable gut injury despite renal injuries. Moreover, Gdf15 was not involved in renal injury aggravation in high fat diet model. Therefore, we could conclude Gdf15 contributed to gut-renal axis pathology in the present study as mentioned in the result. However, it is also valuable to evaluate in other diverse renal injury models such as 5/6-nephrectomixed-mice, adenine, and UUO as suggested by comments.

In the text "*We assessed a representative model of chronic kidney disease via treatment with high fat diet (HFD) (**Supplementary figure S3**). In this model, we could not find notable histological defects in the gut while chronic exposure to HFD (12 weeks) caused renal injuries such as the tubular dilation. However, Gdf15 did not contribute to HFD-induced renal pathological events. On an assumption that gut injury is associated with the adverse outcomes in the kidney, we further determined whether colitis-inducing insult affects renal integrity. Animals with the dextran sodium sulfate (DSS)-induced ulcerative colitis displayed pathological outcomes in the kidney, including elevated BUN levels and tubular dilation, which were notable Gdf15 KO animals (**Fig. 4C-4D).** In addition to tubular distress, we found that Gdf15 deficiency increased glomerular injuries, including morphological shrinkage and neutrophil infiltration in DSS-induced colitis model (**Fig. 4E**).*"

2) Authors investigated the autophagy pathway in the renoprotective and mucosa-protective mechanisms of Gdf15. But only the LC3B protein expression was reported as the marker of autophagy pathway. It should be better to analyze the p62 expression and visualized autophagosome dynamics in cell culture model to confirm the autophagy dynamics in cisplatin-induced HK2/HCT-8 cell culture models. The data of autophagy pathway cisplatin-indued AKI mice and DSS-induced colitis model mice are desirable to confirm the role Gdf15- autophagy

pathway autophagy pathway.

Response: As a reviewer pointed out, we performed additional experiments (**Figure 7A-B, Fig. 8A/9C/9D/9E**) as follows,

- **The autophagic flux**: "*Considering the Gdf15-mediated counteraction against the renointestinal injuries, the autophagy was evaluated as one of representative protection mechanisms. First, we assessed the involvement of Gdf15 in the autophagic pathway in the gut and kidney cells using the plasmid for expression of mCherry- and GFP-tagged microtubule-associated protein 1A/1B light chain 3 B (LC3B), a representative autophagosome marker **(Fig. 7A-7B).** The fusion of autophagosomes to late endosomes or lysosomes results in acidic autolysosomes where the signal of acid-sensitive GFP is lost. Even the signal of acid-insensitive mCherry is ultimately lost when the double tagged protein is degraded. CP-insulted cells exhibited the autophagosome formation (the yellow puncta), which was followed by the autolysosome formation (the red) for clearance of the ubiquitinylated proteins under the chemical stress. However, Gdf15-deficienct cells showed defects in the normal autophagic flux. The autolysosome formation was remarkably interfered in Gdf15 knockdown, resulting in the prolonged yellow puncta signal with attenuated levels of red fluorescence puncta (Fig. 7A-7B), suggesting pivotal roles of Gdf15 in facilitating the autophagic flux.*"

-**p62 in gut cells:** "*Moreover, we observed p62, a receptor for cargo destined to be degraded by autophagy, which binds to ubiquitin and LC3, facilitating clearance of ubiquitinated proteins in the autolysosome. Although p62 was ultimately degraded in CP-exposed cells, Gdf15 deficient cells exhibited resistance to CP-induced degradation process (**Fig. 8A**).*"

-**p62 in kidney cells:** "*Moreover, Gdf15-deficient cells displayed enhanced levels of p62, a receptor for cargo destined to be degraded by autophagy, indicating that Gdf15 contributes to the autolysosome process as well as the autophagosome formation in the renal tubular cells (**Fig. 9D**).*"

-**experimental animals**: "*Experimentally, Gdf15 KO mice exhibited reduced expression of LC3B II, a representative autophagosome marker **(Fig. 9C)**, thereby indicating that Gdf15 positively regulates autophagy.*"

Responses to Reviewer #2' comments

It was my pleasure to read the manuscript "Stress-responsive Growth Differentiation Factor 15 Counteracts Acute Renointestinal Distress via Mucoprotective Reprogramming" by Moon et al. Overall the paper is well written. The topic is important and interesting to the nephrologic community. The paper provides new mechnistical insights into the (patho-)physiological role of gdf15 during renal/intestinal injury.

I have only minor remarks:

1) Because reasons for CKD and AKI and patient cohorts can largely differ, it would be interesting to the reader to have a little bit more information on the analyzed cohorts (GSE30718 and GSE66494).

Response: As a reviewer pointed out, we edited the description in more informative ways as "

2) Sometimes I read GDF15 sometimes gdf15 (e.g. Fig. 1, 3). Please unify.

Response: As a reviewer pointed out, we edited it in more informative ways as
- AKI: *"Renal gene expression was assessed in patients with AKI (gse30718, n = 47) or CKD (gse66494, n = 61). Gene expression analysis of human kidneys with AKI is limited since such kidneys are seldom biopsied [66]. Given the limited supply of kidney biopsy samples from patients with AKI, patients with kidney explants were followed u p as models of human AKI [66]. Since all subjects with kidney transplants experience A KI, early kidney transplants without rejection are an excellent model for human AKI. Biopsies from transplants with AKI (gse30718) were compared with those from the pr istine protocol biopsies of stable transplants.*
- CKD: *"Patients with CKD displays the irreversible and progressive loss of nephrons, followed by the glomerular fibrosis, tubular atrophy, and tubulointerstitial fibrosis, whi ch are commonly observed features of human progressive renal diseases, irrespective of the initial etiologies [67]. Biopsy specimens from 48 patients with histopathologically confirmed CKD (gse66494) were analyzed to identify genes responsible for tubulointer stitial fibrosis and tubular cell injury using two independent discovery and validation processes [67]. The patients exhibited various histological types of renal diseases includi ng IgA nephropathy (n = 15), membranous nephropathy (n = 7), lupus nephritis (n = 6), minimal change nephrotic syndrome (n = 3), membranoproliferative glomerulone phritis (n = 3), amyloidosis (n = 3), antineutrophil cytoplasmic antibody-related glom erulonephropathy (n = 2), diabetic nephropathy (n = 2), and other nephropathies (n = 6)."*

3) Fig. 2 legend is mixed up 2B -> 2C etc. Moreover, not all parts of Fig. 2 are covered by the legend.

Response: As a reviewer pointed out, we improved all legend 2 in a correct way.

4) The criteria used (grading) for the histological grading (2F-H) are not clear to me.

Response: As a reviewer pointed out, we improved the pathological scoring section as follows, "*The degree of renal tubular injury was expressed as the proportion of damaged renal tubules. For statistical analysis, visual fields of each specimen were randomly selected, and tubule diameters and lumen diameters were photographed under the microscope, and the lesions were statistically analyzed by ImageJ software. Tubule diameters were measured from the basal membrane across the narrowest segment of a tubule to the basal membrane of the opposite side. Lumen diameters were determined by measuring the distance between the apical membranes across the narrowest portion of a tubule. The levels of the tubular vacuolization were assessed by grading levels of vacuoles affecting proximal tubules and extending to basement membrane. The cystic index was calculated as the percentage of cystic area relative to the total kidney area.*"

5) Fig 7A is the number under HCT-8 control (+) row 12.28 or 2.28?

Response: As a reviewer pointed out, we edited the values in the **figure 8A**.

REVIEWERS' COMMENTS:

Reviewer #1 (Remarks to the Author):

In this study, Ray and colleagues examined a role of Growth differentiation factor 15 (Gdf15) in stress response mechanisms of renal injury and intestinal mucosal injury.They performed trnscriptome analysis of patients with acute kidney injury (AKI) or chronic kidney disease (CKD) and identified protein kinase R (PKR) positively associated with Gdf15 expression in patients with renal injury.
They utilized three animal models, cisplatin-indued AKI mice, DSS-induced colitis model mice and high fat diet mice with wild type mice and Gdf15 deficient mice and demonstrated protective effects of Gdf15 in renointestinal distress.accessed kidney injury in both cisplatin-indued AKI mice and DSS-induced colitis model mice.
HK2 cells and HCT-8 cells were also used in cell culture model of the kidney and the gut, respectively.Cisplatin treatment increased Gdf15 expression in both HK2 cells and HCT-8 cells in stress-related mammalian eIF2α kinase, i.e. protein kinase R(PKR) dependent manner. They reported one of the protective mechanism of Gdf15 was associated autophagy. They assessed the involvement of Gdf15 in the autophagic pathway in the gut(HCT8) and kidney(HEK293) cells using the plasmid for expression of mCherryand GFP-tagged microtubule associated protein 1A/1B light chain 3 B (LC3B), a representative autophagosome marker and reported disrupted autophagy flux in gdf-15 deficient mice. Also authors observed both LC3b and p62 in the kidney cells(HK2) and the gut cells(HCT8). Finally, authors added the experiment of LC3BI, LCDBII and p62 protein expression in mouse kidney from wild type-control mice, cisplatin-AKI wild type mice and cisplatin-AKI/gdf-15 Ko mice.

Reviewer #2 (Remarks to the Author):

Thanks for addressing my questions and points raised.
